# Mitochondria-Targeted SmsHSP24.1 Overexpression Stimulates Early Seedling Vigor and Stress Tolerance by Multi-Pathway Transcriptome-Reprogramming

**DOI:** 10.3389/fpls.2021.741898

**Published:** 2021-11-23

**Authors:** Muslima Khatun, Bhabesh Borphukan, Iftekhar Alam, Chaman Ara Keya, Varakumar Panditi, Haseena Khan, Saaimatul Huq, Malireddy K. Reddy, Md. Salimullah

**Affiliations:** ^1^Plant Biotechnology Division, National Institute of Biotechnology, Dhaka, Bangladesh; ^2^Crop Improvement Group, International Centre for Genetic Engineering and Biotechnology, New Delhi, India; ^3^Department of Biochemistry and Microbiology, North South University, Dhaka, Bangladesh; ^4^Department of Biochemistry and Molecular Biology, University of Dhaka, Dhaka, Bangladesh; ^5^Molecular Biotechnology Division, National Institute of Biotechnology, Dhaka, Bangladesh

**Keywords:** eggplant, mitochondria, chaperon, abiotic stress, heat tolerance, small heat shock protein

## Abstract

Among the diverse array of heat shock proteins across the three domains of life, mitochondria-targeted small heat shock proteins (sHSPs) are evolved in the plant lineage. However, they remained mysterious and understudied. In this study, we reported a systematic study of a novel mitochondria-targeted nuclear sHSP from eggplant (*Solanum melongena* L.; SmsHSP24.1). Differential expression of *SmsHSP24.1* indicated its positive role exerted during stress conditions. *Escherichia coli*-BL21 cell line overexpressing the *SmsHSP24.1* showed excellent thermo-tolerance ability, tolerating up to 52°C. Spectrometry and electron microscopy revealed a multimeric structure of the protein which acted as a molecular chaperone at high temperatures. Overexpression of *SmsHSP24.1* significantly enhanced resistance against heat, drought, and salt stresses and showed rapid germination in constitutively overexpressed eggplant lines. RNA-seq analysis reveals an apparent upregulation of a set of reactive oxygen species (ROS) scavenging enzymes of the glutathione (GHS) pathway and mitochondrial electron transport chain (ETC). Significant upregulation was also observed in auxin biosynthesis and cell-wall remodeling transcripts in overexpressed lines. qPCR, biochemical and physiological analysis further aligned with the finding of transcriptome analysis and suggested an essential role of *SmsHSP24.1* under various stress responses and positive physiological influence on the growth of eggplants. Therefore, this gene has immense potential in engineering stress-resilient crop plants.

## Introduction

Environmental factors continuously influence the performance of agronomic traits. Heat stress, among others, is one of the significant regulating factors that influence the metabolic pathways, growth, and development of crop plants and ultimately reduces the yield. Heat stress also imbalance the delicate cellular homeostasis, which turns down total protein synthesis, stability, and activity (Mayer and Bukau, 2005). This deregulation of cellular homeostasis activates a chain of events, such as instability of cell membranes and altered osmosis, a sudden increase in the concentration of reactive oxygen species (ROS), etc., which leads to organelle malfunction, imbalance in phytohormones production and signaling, and reprogramming of transcriptomic and metabolic pathways, thereby limiting the growth and productivity of plants (Hasanuzzaman et al., 2013). With increasing global temperature, heat stress poses severe threats for crop production, including eggplant (*Solanum melongena*) in the Indian subcontinent. It is estimated that across South Asia, the temperature would rise by approximately 2.2°C annually, which will cause significant damage to the performance of the eggplant in the coming years (Hoegh-Guldberg et al., 2018). Therefore, studying the impact of high temperature (HT) on the growth and development of crop plants is vital to maximizing agricultural production and ensure food security, especially for the resource-poor farmers in the South Asian region whose livelihoods directly depend on agriculture. Thus, it is important to understand the mechanism behind heat stress tolerance in eggplants.

Throughout the evolution, plants developed several stress tolerance mechanisms as they continuously expose to different stresses including heat (Suzuki, 2016). To counter this, plants have evolved diverse mechanisms executed at varying levels of metabolomics or proteomics pathways. Small heat shock proteins (sHSP; 12–42 kD molecular weight) are considered one such important protective mechanism, which is active in all living organisms, including plants. There are a total of 12 diversified subfamilies of sHSP present ubiquitously in three domains of life (Sun et al., 2020). Among all the subfamilies of sHSPs, the mitochondria localized sHSPs (Mito-sHSP) are evolved exclusively within the plant lineage (Waters and Vierling, 2020) with rare exceptions in *Drosophila* HSP22 (Morrow et al., 2000). Also, previous studies showed that plants tend to accumulate more mitochondrial small heat shock protein compared with HSP60 and HSP70 under heat stress conditions (Vierling, 1991; Lenne and Douce, 1994).

It is well known that mitochondria are the source of ATP production and also lead to ROS formation under adverse environmental conditions (Zhang et al., 2009; Huang et al., 2016). However, cellular ROS has both positive and negative effects on growth and development based on the concentration gradient (Bailly et al., 2008). Previous studies have demonstrated the relation of cellular ROS gradient and availability of small HSPs during stress conditions by protecting ROS scavenging enzymes (Kong et al., 2014). Higher seed vigor, longevity, and seedling establishment were achieved by overexpressing *OsHSP18.2* in *Arabidopsis* (Kaur et al., 2015). Higher rates of germination and elongated hypocotyls were observed in *CsHSP17.2* overexpressed *Arabidopsis* (Wang et al., 2017). Similarly, transcriptional reprogramming due to overexpression of small HSPs involved in the different biological pathways has also been reported in creeping bentgrass (Sun et al., 2016). However, a similar role of Mito-sHSP is still emerging in plants compared with other cytosolic sHSPs. It has been well documented that *Drosophila* Mito-sHSP22 is involved in protecting mitochondrial protein homeostasis and plays a crucial role in aging regulation and is essential for longevity (Morrow et al., 2004; Morrow and Tanguay, 2015). This shows the importance of mitochondria localized sHSP proteins under both normal and stressed conditions. A recent study on cotton shows that under heat stress, Mito-sHSP24.7 promotes rapid germination by blocking electron transport at cytochrome c, leading to enhanced ROS, which facilitate seed testa rupture and early germination (Ma et al., 2019). Another study on apple has demonstrated that mitochondrial sHSP protects the first protein complex of the electron transport chain NADH (ubiquinone oxidoreductase) during heat stress (Downs and Heckathorn, 1998). The study of Sanmiya et al. (2004) revealed that overexpression of tomato mitochondrial sHSP23.8 protein in transgenic tobacco lines shows higher thermotolerance, whereas tobacco plants that carry antisense of sHSP23.8 mRNA were susceptible to heat. Similarly, overexpression of sHSP23.8 in transgenic tomatoes shows increased thermotolerance in both T0 and T1 progenies under heat shock conditions (Nautiyal et al., 2005).

All these pieces of evidence suggest a crucial role of mitochondria localized nuclear small heat shock protein in protecting plants from various stresses. To date, compared with other subfamilies of heat shock proteins, only a few Mito-sHSPs are studied to understand their physiological or stress-protective role, such as pea (Lenne et al., 1995; Avelange-Macherel et al., 2020), tomato (Banzet et al., 1998; Liu and Shono, 1999), maize (Lund et al., 1998, 2001), rice (Mani et al., 2015), Cotton (Ma et al., 2019), and *Arabidopsis* (Scharf et al., 2001; Hooper et al., 2017; Escobar et al., 2021). Therefore, much of the function of mitochondria localized sHSPs and their potential role in plant growth and development remain largely unknown. In this study, we had investigated the physiological functions of a mitochondria-targeted small HSP (Mito-*SmsHSP24.1*) along with its protective role under stress conditions from a novel source which was the eggplant. In order to understand the impact of Mito-*SmsHSP24.1* under normal and stressed conditions, we had developed transgenic eggplants that constitutively overexpressed Mito-*SmsHSP24.1*. We had systematically studied its effect on growth and development under normal and stressful conditions. Transcriptome profiling indicated global reprogramming in crucial paths which led to significant alteration of crucial agronomic traits, specifically in early germination and seedling vigor in transgenic lines. Our results revealed a novel source of mitochondria localized sHSP protein, which could be beneficial for engineering climate-resilient crops in the future.

## Materials and Methods

### Plant Material and Growth Conditions for Stress Treatments

We used eggplant (*S. melongena*) variety BARI Begun-4 which was developed by the Bangladesh Agricultural Research Institute (BARI), Joydebpur, Gazipur, Bangladesh. Specifically, 3-week old seedlings were grown hydroponically in Yoshida nutrient solution (YS; HIMEDIA, Mumbai, India) and subjected to different stresses like heat (45°C), salt (200 mM NaCl), and drought (150 mM) mannit to understand the response of *SmsHSP24.1* expression in leaf tissues. Plants irrigated with water served as treatment controls (CT). Eggplant leaves were collected at 0, 1, 2, 4, 6, 12, and 24 h after stress treatment and used for RNA isolation followed by subsequent gene expression analysis.

For physiological analysis of *SmsHSP24.1* overexpressing transgenic eggplant lines, three different southern positive lines (OE1, OE3, and OE7) were used under both normal and stress conditions. Specifically, 2-week old seedlings of OE1, OE3, and OE7 lines along with wild-type plants (WT) shifted in soil pots were used for heat and drought stress experiments, and seedlings transferred to Yoshida nutrient solutions were used for salt stress. After 1 week of adaptation, seedlings were exposed to heat shock (45°C) for up to 36 h. Yoshida nutrient solution was supplemented with 200 mM NaCl for salt treatment for 12 h and as drought treatment, water was withheld for 10 consecutive days to study the growth parameters of transgenic lines (OE) compared with the control (WT). Similarly, after combined heat (45°C) and dt stress by withdrawal water for 25 days at ICGEB net house field, leave tissues were collected. These samples were used to study oxidative damaged, biochemical and enzymatic assays as well as expression profiling of the target transcripts, i.e., *NADH-ubiquinone oxidoreductase*, q*uinol-cytochrome c- oxidoreductase*, *aminopeptidase* (*PepA*), *auxin-responsive gene* (*SAUR*), *superoxide dismutase* (*SOD1, SOD2*), *catalase* (*CAT*), and *ascorbate peroxidase* (*APX*) transcript by qRT-PCR.

### Extraction of Plant Genomic DNA, RNA, and qRT-PCR Analysis

Genomic DNA was extracted from leaves of 21-days-old eggplant seedlings using the PureLink Genomic Plant DNA Purification Kit (Invitrogen^1^, United States) according to the instructions of the manufacturer. Total RNA was extracted from eggplant tissues using the PureLink Plant RNA Reagent kit (Invitrogen; see text footnote 1). RNA was treated with RNase-free DNase (NEB^2^, Ipswich, MA, United States) and used for first-strand cDNAs synthesis according to Verso cDNA Synthesis Kit (Thermo Fisher Scientific, United States; see text footnote 1). Real-time qRT-PCR was performed with SYBR Green PCR Master Mix and Applied Biosystems in 7500 Real-Time PCR Systems (Thermo Fisher Scientific, United States; see text footnote 1). Eggplant 18S rRNA-specific primer pair was used as an internal reference gene. All primer pairs used for RT-PCR and qRT-PCR are mentioned in Supplementary Table 2.

### Sequence Identification, Isolation of cDNA, Phylogenetic Analysis, and Homology Modeling of Novel SmsHSP24.1 Protein

The putative eggplant mitochondria localized nuclear *SmsHSP24.1* gene was identified using NCBI expressed sequence tags (ESTs) database (Supplementary Figure 1A). The full-length *SmsHSP24.1* cDNA was amplified by nested PCR using a high-fidelity DNA polymerase (KOD plus, Toyobo, Japan) with primer pairs of *SmsHSP24.1*_F1/R1 and F2/R2 (Supplementary Table 2) and cloned into PCR-4-TOPO vector (Invitrogen, United States). Sequence confirmation was done using universal M13 forward and reverse primers through the Macrogen sequencing platform (Seoul, Republic of Korea) (macrogen.com/ko). MODELLER 9 version 11 (Sali et al., 1995) program was used for homology modeling of SmsHSP24.1 against Mito_AtHsp21 (Protein databank ID P31170) (Supplementary Figure 1B). Evaluation tools ProCheck (Laskowski, 1993) and Verify3D (Eisenberg et al., 1997) were applied to assess the predicted three-dimensional model of SmsHSP24.1 protein. The Muscle (Edgar, 2004) program was used for multiple sequence alignment, and a phylogenetic tree was constructed using MEGAX (Kumar et al., 2018) according to the neighbor-joining method with bootstrap value 1,000 (Supplementary Figure 1C).

### Subcellular Localization of mGFP-Fused SmsHSP24.1 Protein in Tobacco Leaves and Eggplant Cell Culture

Bioinformatics-based prediction tools TargetP^3^ (Denmark) and MitoFates (Fukasawa et al., 2015) were used to check the SmsHSP24.1 protein targeting signal (Supplementary Figure 2). To further *in vivo* confirmation, *SmsHSP24.1* protein-coding sequence without stop codon was cloned into mGFP based pCAMBIA1302 expression vector with hygromycin selection. *Bgl*II and *Spe*I restriction enzymes were used for the cloning of this sequence (Supplementary Figures 3A-C). Primers used for cloning confirmation are enlisted in Supplementary Table 2. *Agrobacterium* strain EHA105 was used for transient expression of the final expression cassette in tobacco leaf epidermal cells by following the method described by Kokkirala et al. (2010). After 72 h of inoculation, the leaf tissues were observed under a confocal fluorescence scanning microscope (Zeiss LSM510, Germany).

Similarly, stably integrated eggplant cell suspension culture with the same construct was used to further confirmation by co-localization with mitochondria specific dye MitoTracker^TM^ Red FM (Cat no: M22425; Invitrogen, United States) following a method similar to the one developed in the crop improvement lab of Reddy (data unpublished, ICGEB). Samples were prepared by adding 10 μl of suspended cells on a slide, and photographs were taken by laser scanning confocal microscopy (Zeiss LSM510, Germany). The fluorescence signal was collected using an emission filter of a 500–535 nm bandpass for GFP and 581–644 nm bandpass for MitoTracker^TM^ Red FM. All fluorescence experiments were independently repeated at least three times.

### Construction of Binary Expression Vectors and Generation of Overexpression Lines

The *SmsHSP24.1* coding region (*Nde*I + *Not*I) was first cloned into Gateway compatible entry vector (pL12R34-Ap) under constitutive cauliflower mosaic virus promoter (CaMV 35S) (*Kpn*I + *Nde*I) and nopaline synthase terminator (Nos) (*Not*I + *Sac*I) and then transferred into the plant transformation vector pMDC100 (Invitrogen, United States) containing *nptII* (kanamycin-resistant) gene as a plant selection marker (Supplementary Figures 3D-G). *Agrobacterium* strain, EHA105 was used for plant transformation, and 21-days-old cotyledonary leaves were used as starting material. Kanamycin (100 mg/l) was used at a 10-day interval to select transformed lines. The putative transgenic lines were screened by PCR using *nptII* and *SmsHSP24.1* gene-specific primers. For Southern blot analysis of putative transgenic lines, approximately 20 μg genomic DNA was digested with the restriction enzyme, *Nde*I. DNA fragments were separated on a 0.8% agarose gel and blotted onto the Hybond^TM^ N^+^ nylon membrane (GE Healthcare Limited, United Kingdom). Subsequently transferred N^+^ nylon membrane was hybridized with a 500 bp of *SmsHSP24.1* gene-specific DIG-labeled probe (PCR DIG Probe Synthesis Kit, Roche, Germany). The blot was then washed and detected according to the instructions of the manufacturer (DIG High Prime DNA Labeling and Detection Starter Kit II, Roche, Germany).

### Recombinant Protein Production, Thermal Stability, and Protein Solubility Assay

The protein-coding sequence *SmsHSP24.1* was cloned between *Nde*I and *Not*I restriction sites of pET28a vector (Supplementary Figures 3H-K; Primers enlisted in Supplementary Table 2) for recombinant protein production in *E. coli* BL21 (DE3) cells. 1 mM of isopropyl-β-D-thiogalactopyranoside (IPTG) was used for induction at different temperatures (37, 28, and 18°C) and analyzed by sodium dodecyl sulphate polyacrylamide gel electrophoresis (SDS–PAGE).

For studying the thermotolerance capacity of SmsHSP24.1 protein, we followed a method similar to the one described by Wang et al. (2017). *Escherichia coli* harboring pET28a:SmsHSP24.1 and pET28a blank plasmid were used for the assay at 37, 42, 48, 52, 55, and 58°C, respectively. Cell viability was estimated by counting the number of colony-forming units. Parallelly total soluble protein was also analyzed from supernatants and pellets in SDS–PAGE, visualized by Coomassie Blue staining.

### Chaperone Like Activity Assay of SmsHSP24.1

For the chaperone-like activity assay, thermal-induced aggregation of alcohol dehydrogenase (ADH) (50°C) and citrate synthase (CS) (45°C) were measured in the presence or absence of SmsHSP24.1 protein at various molar ratios, according to Ihara et al. (1999). Protein aggregation was monitored by measuring light scattering at 360 nm in three replicates for each treatment. The measurements were performed using Helios Gamma Spectrophotometer (Thermo Spectronic, Cambridge, United Kingdom). For further investigation, electron microscopic analysis was performed with purified SmsHSP24.1 protein and the model substrate, citrate synthase (CS) before and after thermal-induced aggregation according to Zhang et al. (2015). Electron micrographs were recorded with a JEOL 1400 transmission electron microscope operated at 100 kV in ICGEB, India facility.

### Seed Germination Assay

Seed germination assay of WT and OE lines was done under normal and abiotic stress conditions. MS growth medium was supplemented with 200 mM NaCl for salt stress and 150 mM mannitol for drought stress. For heat stress, seeds were first kept in an incubator at 45°C heat for 2 and 4 h then placed in full strength MS growth medium. A growth room condition of 16 h photoperiod at 25 ± 2°C temperature was provided. Germination rates were calculated each day, up to 7-day incubation, and representative seedlings were photographed.

### Measurements of Chlorophyll Content and Physiological Parameters

Total chlorophyll content of OE and WT seedlings under untreated and stressed conditions were measured using a spectrophotometric method described by Lichtenthaler and Wellburn (1976) with slight modification. Approximately 150 mg of leaf tissue was used for chlorophyll measurement, with each reaction was performed in three replicates. The absorbance was measured at 441, 646, 652, and 663 nm using a Helios Gamma Spectrophotometer (Thermo Spectronic, Cambridge, United Kingdom). Physiological parameters, namely plant height (cm), shoot length, root length, fresh weight, and dry weight of transgenic and WT lines were measured under normal and abiotic stress conditions.

### Biochemical and Enzymatic Activity Assays of SmsHSP24.1 Protein Overexpressed Lines

Transgenic lines and WT plants from untreated and stressed conditions were harvested for assay of antioxidant enzyme activity. Approximately, 200 mg of fresh leaves were finely ground in ice-cold 0.2 M phosphate buffer (pH 8) and centrifuged at 15,000 rpm for 15 min at 4°C. The supernatant was kept on ice. The superoxide dismutase (SOD) activity was performed as previously described by Beyer and Fridovich (1987). Absorbance was measured at 560 nm Ascorbate peroxidase (APX) activity was determined by following dihydrogen dioxide (H_2_O_2_) dependent oxidation of ascorbic acid (ASC) at 290 nm. The assay for catalase (CAT) activity was performed according to the protocol of Beaumont et al. (1990), which monitored the dismutation of H_2_O_2_ at 240 nm. Electrolyte leakage was measured as previously described (Sairam et al., 2002). Measurement of proline was also performed according to the protocol of Bates (1973).

### Oxidative Damage and Dead Cell Detection Assay

Leaf discs from both WT and transgenic plants of similar age were treated with 10 μM methyl viologen (MV; paraquat) for oxidative damage, 200 mM NaCl as salt stress, and 150 mM mannitol as drought stress. For MV stress, leaf discs were incubated for 6 h at 28°C dark in a shaker incubator and then exposed to light. Salt and drought-imposed leaf discs were observed after 72 h of incubation under the light. Generation of H_2_O_2_ was detected with 3, 3-diaminobenzidine (DAB) as previously reported by Thordal-Christensen et al. (1997). *In vivo* generation of O^–2^ in tissues after applying the stresses mentioned was detected by staining with 1% nitro blue tetrazolium (NBT) described by Fryer et al. (2002). Similarly, cell death was also detected in treated samples after staining with lactophenol trypan blue as described by Keogh et al. (1980).

### RNA-Seq and Transcriptome Analysis

Total RNA was extracted from the leaves of 3-week-old WT and T2 generation transgenic eggplants (OE7) under either normal or heat stress (45°C) conditions using the RNAeasy Plant Mini Kit (Qiagen^4^, Germany) according to the instructions of the manufacturer. The quality and quantity of RNA were confirmed by RNase-free agarose gel electrophoresis and a Bioanalyzer (Agilent Technologies, Santa Clara, CA, United States). Library construction and RNA sequencing were carried out by Agri-Genome Labs, Kerala, India. Only samples with RNA integrity of N7.0 were used for RNA-seq analysis. Illumina HiSeq 2500 platform (Illumina Inc., CA, United States) was used for RNA sequencing. All the RNA-seq data were then successfully submitted to the NCBI SRA database (PRJNA750594). The pre-processed and rRNA removed reads were aligned to the tomato reference genome, and the gene model downloaded from Sol Genomics Network^5^. After aligning the reads with the reference genome, the aligned reads were used to estimate genes and the expression of transcripts using the cufflinks program (version 2.2.1, United States). Differential expression analyses of all control and treated samples were performed using the cufflinks program. Levels of gene expression were measured by the fragments per kilobase of transcript per million fragments mapped (FPKM) plot. Log2 fold change cut-off 2 and *p*-value cut-offs.01 and.05 were used separately as cut-offs for up and down-regulated genes and isoforms. Clustered heat maps of up- and down-regulated genes of sample comparison with a *p*-value cut-off of.05 were generated. Gene ontology (GO) enrichment analysis was performed using the GOSeq tool (default parameters; United States). GOseq functions in calculating the significance of over-representation of each GO category amongst differentially expressed genes (DEGs). The KEGG pathway analysis was carried out for the available differentially expressed genes using a bio-conductor tool, path view with the tomato reference genome.

### Statistical Analysis

Statistical analyses were performed by one-way ANOVA and the differences between means were compared with Tukey HSD.5 values obtained for the particular dataset.

## Results

### Identification, Cloning, and Sequence Analysis of *SmsHSP24.1* Gene From Eggplant

The putative 1032 bp gene was retrieved by NCBI ESTs blast using the tomato Mito-*slHSP23* (BAA32547.1) full-length cDNA as a query. *In silico* sequence analysis using eggplant draft genome database (Hirakawa et al., 2014), we found one copy of mitochondria-targeted *small HSP* gene at Sme2.5_00899.1_g00005.1 contig with two exons and an open reading frame (ORF) of 636 bp. We amplified the *SmsHSP24.1*-ORF with nested PCR primers that encode a protein with an apparent molecular weight of 24.1 kDa and an isoelectric point of (pI) 4.84. Blastp homology search and multiple sequence alignment and homology modeling indicated that SmsHSP24.1 contained a conserved α-crystalline domain (ACD) of 81-amino-acid at the C-terminus (positions + 115 to + 196). Phylogenetic analysis with the deduced amino acid sequences from a diverse range of species revealed that this protein was closely related to tomato (SlsHSP23.5) and *Arabidopsis* (AtsHSP26.4). Therefore, we designated our protein as SmsHSP24.1 and submitted it to the NCBI gene bank (AXS76128.1).

### SmsHSP24.1 Response to Multiple Environmental Stresses

**FIGURE 1:**
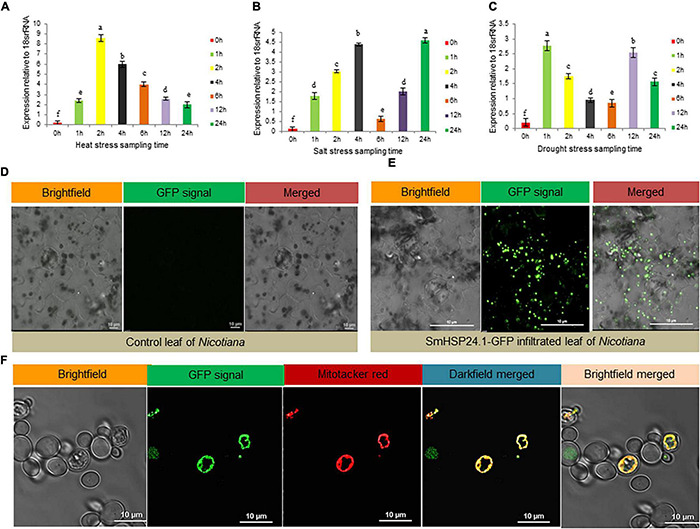
Stress-responsive expression analysis and subcellular localization of SmsHSP24.1 protein. **(A)** Time-dependent relative quantitative RT-PCR analysis of 3-week-old eggplant seedlings grown in soil pot at 45°C heat. **(B,C)** Relative RT-PCR analysis of seedlings grown hydroponically in Yoshida nutrient solution containing 200 mM NaCl salt and 150 mM mannitol. RT-qPCR data are *M* ± *SD* (*n* = 3). The letters a, b, c, and others indicate significant differences between samples (*P* < 0.05). **(D)** Fluorescence scanning confocal microscopy of the Agro-infiltrated control tobacco epidermal cells appeared with no mGFP fluorescent signal. **(E)** Agro-infiltrated tobacco epidermal cells with pCAMBIA:CAMV35SP:SmsHSP24.1-mGFP:NosT construct revealed punctate structures in leaf epidermal cells which appeared to be localized in the mitochondria. **(F)** Stably transformed eggplant suspension cells with pCAMBIA:CAM35SP:SmsHSP24.1-mGFP:NosT construct were directly observed under a confocal microscope with co-localization of mitochondria specific dye MitoTracker^TM^ FM Red. The bar represents 10 μm.

We had investigated *SmsHSP24.1* transcript abundance to understand its physiological role under abiotic stress conditions. The level of *SmsHSP24.1* transcript in leaves under heat (45°C), salt (200 mM NaCl), and osmotic stress (150 mM mannitol) were compared with untreated seedlings as relative fold-change (Figures 1A-C). The relative fold change of *SmsHSP24.1* under heat stress was found to be the highest among all the treatments. An elevated transcript level was observed within 1 h after the heat treatment which reached a maximum of 9-fold change at 2 h time point and then gradually declined (Figure 1A). In the case of salt stress, the expression dynamics of the *SmsHSP24.1* transcript were found to be different from the heat stress. An approximately 5-fold increase of *SmsHSP24.1* transcript was observed at the 4 h time point of salt stress after which it reduced gradually until 12 h and then a significant upregulation was observed again at the 24 h timepoint (Figure 1B). A quick expression of *SmsHSP24.1* was witnessed when treated with mannitol reaching a peak as soon as this osmotic agent was added to the plant. However, after 2 h the level of *SmsHSP24.1* transcript started to decline steadily up to 12 h and at 24 h, it was 1.5-fold lower than the level transcript observed at 1 h of drought stress (Figure 1C).

### SmsHSP24.1 Is a New Member of Mitochondria Localized Nuclear-Encoded Protein

*In silico* analysis using TargetP (see text footnote 3) and MitoFates (Fukasawa et al., 2015) revealed that the *SmsHSP24.1* protein was localized in mitochondria with a significantly high score of 0.792 and 0.997, respectively. Similarly, *in vivo*, transient expression assay of Agrobacterium harboring *SmsHSP24.1* fused with mGFP on tobacco leaf (3-week-old seedlings) also indicated discreet localization other than the nucleus (Figures 1D,E). Furthermore, co-localization analysis with mitochondria specific dye (MitoTracker^TM^, United States) FM (Cat no: M22425; Invitrogen) in eggplant cell suspension-culture conclusively confirmed that this novel SmsHSP24.1 was targeted to the mitochondria (Figure 1F).

### Heterologous Overexpression of SmsHSP24.1 Protein Acts as a Molecular Chaperon and Enhances Thermo-Tolerance of *E. coli* Cells Under Severe Heat Stress

**FIGURE 2:**
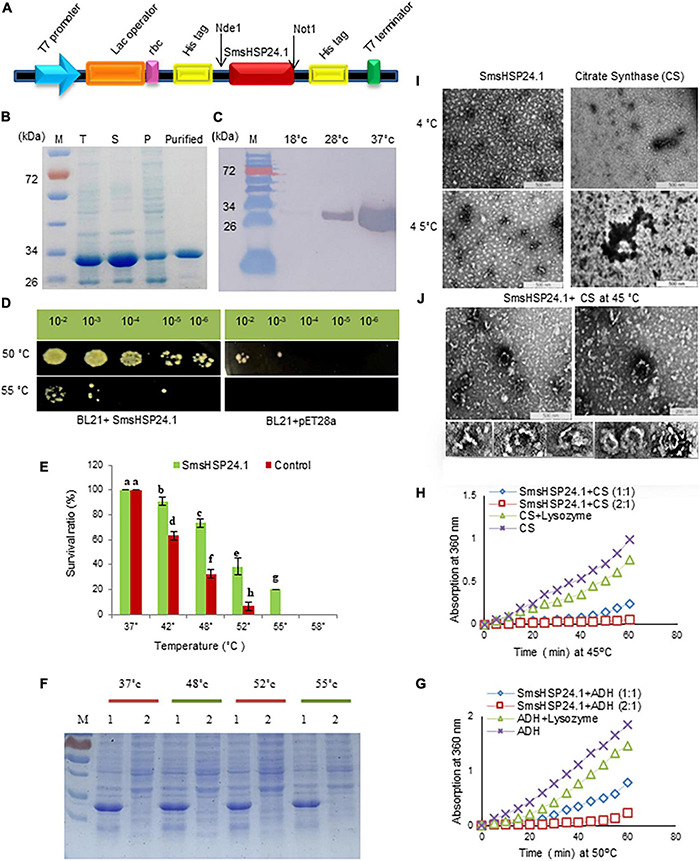
Recombinant SmsHSP24.1 protein expression and chaperone activity assay. **(A)** Schematic diagram of SmsHSP24.1 protein expression cassette. **(B)** SDS–PAGE analysis of the proteins present in the total lysates (T), soluble (S), insoluble pellet (P), and purified SmsHSP24.1 protein. **(C)** Western blot of SmsHSP24.1 protein with Anti-His tag antibody. **(D)** pET28a:SmsHSP24.1 and control pET21a plasmid harboring BL21 cells grown in LB medium after being heat-shocked for an hour at 50°C and 55°C. **(E)** Colony-forming units counted from heat-treated and non-treated cells at different temperatures. **(F)** Proteins solubility was analyzed in SDS–PAGE at different temperature periods; 1: BL21a:SmsHSP24.1, 2: Blank BL21a. **(G,H)** Thermal aggregation of CS and ADH detected by light scattering at 360 nm. **(I)** Transmission electron microscopy images of SmsHSP24.1 and citrate synthase (CS) protein at 4°C and 45°C, respectively, Scale bars represent 500 nm. **(J)** Electron microscopy images clearly show SmsHSP24.1 protein prevents model substrate CS when incubated at 45°C for 60 min. Scale bars represent 500 nm (Left panel) and 200 nm (Right panel), SmsHSP24.1protein forming different polyhedron assemblies (Lower panel).

We performed a series of experiments to analyze whether the novel SmsHSP24.1 protein can function as a chaperone and enhance survivability under severe stress conditions. First, we had expressed the coding sequence of SmsHSP24.1 protein in the BL21(DE3) strain of *E. coli* using the expression vector pET28a. Western blot analysis with an anti-His-tag antibody demonstrated the presence of the recombinant protein in the *E. coli* cells (Figures 2A-C). A Thermotolerance assay was conducted with a pET28a vector carrying *SmsHSP24.1* and blank pET28a as an experimental control (Figure 2D). No growth difference was observed under normal growth conditions, but 65% of *E. coli* culture carrying pET28a-SmsHSP24.1 construct found to be effectively tolerated temperature up to 55°C for 1 h, whereas the control cells failed to survive (Figure 2E). Only 10% of the cells carrying the blank pET28a at 50°C survived 1 h compared with 50% of *E. coli* carrying the pET28a-SmsHSP24.1 construct. SDS–PAGE analysis of the soluble fractions further revealed a significant increase in total soluble proteins in the pET28a-SmsHSP24.1 carrying BL21 cells compared with pET28a-blank at different temperatures (Figure 2F).

To further understand, we conducted a transmission electron microscopic thermal-aggregation assay of two commonly used heat-sensitive model substrate proteins, ADH, and CS, at different molar ratios of SmsHSP24.1. Thermal inactivation and aggregation of CS and ADH were detected by light scattering at 45°C and 50°C, respectively (Figures 2G,H). SmsHSP24.1 delayed aggregation of CS at a molar ratio of 1:1 (SmsHSP24.1:CS), and at a 2:1 molar ratio, it fully protected CS from aggregation. Similarly, the aggregation of ADH was also found to be entirely prevented at a 2:1 (SmsHSP24.1:ADH) molar ratio. Transmission electron microscopic (TEM) analysis further showed CS is in its native state at 4°C, whereas it was completely denatured and aggregated at 45°C without the presence of SmsHSP24.1 (Figure 2I), On the other hand, while incubated with SmsHSP24.1, the formation of polyhedron assembly under TEM was observed which correlates with the thermal protection of CS at 45°C up to 60 min (Figure 2J).

### Generation of Constitutively Overexpressed SmsHSP24.1 Eggplant

To further evaluate the physiological role of SmsHSP24.1, we developed independent transgenic eggplant lines constitutively overexpressing SmsHSP24.1 protein (Supplementary Figure 4A). PCR screening with two sets of primers (*NptII* primer sets ∼0.45 kb and SmsHSP24.1 + NosT terminator junction primer set ∼0.9 kb) found 17 out of 20 putative T0 plants to be positive (Supplementary Figure 4B). Homozygous T2 OE lines were selected under 100 mg/L kanamycin and reconfirmed by PCR (Supplementary Figures 4C,D). Same lines were further used for Southern blot analysis to confirm the copy number of transgene integration using the *SmsHSP24.1* gene-specific probe. We obtained two single, two double, one triple, and one multi-copy (containing five copies of insertions) transgenic events with morphological parameters similar to WT eggplants (Supplementary Figure 4E). Similarly, the copy number of Mito-sHSP (*SmsHSP24.1*) in the WT eggplants genome was also confirmed by Southern blot analysis (Supplementary Figure 4F). qRT-PCR analysis showed a higher level of transgene expression compared with the WT (Supplementary Figure 4G). Henceforth, we chose single copy integrated transgenic lines (OE1, OE3, and OE7) with high transgene expression from the T2 generation for subsequent physiological analysis.

### SmsHSP24.1 Overexpression Exerts Improved Tolerance to Abiotic Stresses

**FIGURE 3:**
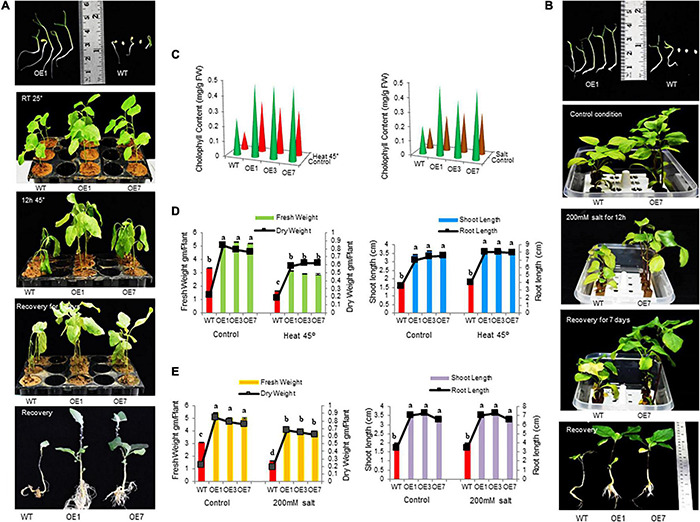
Growth parameters of transgenic eggplant lines overexpressing SmsHSP24.1 protein under heat and salt-stressed conditions. **(A)** Growth parameters of transgenic (OE) as well as WT eggplant lines at 45°C. **(B)** Growth parameters under 200 mM NaCl salt stress condition of the transgenic (OE) and WT eggplant lines. **(C–E)** Total fresh weight, dry weight, root length, shoot length, and chlorophyll contents were significantly higher in all transgenic seedlings compared with the WT line. Data are M ± SD (*n* = 3). The letters a, b, c, and others indicate significant differences between different lines (*P* < 0.05).

Transgenic (OE1, OE3, and OE7) and WT plants were tested under normal and stress conditions. Both grew well and produced new leaves in the untreated condition. However, upon heat stress (45°C) for 36 h, OE lines exhibited marked thermotolerance compared with WT (Figure 3A) and wholly recovered when the stress was withdrawn. During drought stress induced by withholding water for 10 days, WT plants exhibited severe wilting, and some died, while only minor signs of dehydration were observed in OE lines. After 1 week of re-watering, 80% of the OE lines revived compared with 30% WT plants (Supplementary Figure 5). Also, when seedlings were treated with 200 mM NaCl for 3 days, WT plants either exhibited significant growth inhibition or died compared with OE lines. It was also observed that seedlings of OE lines were less prone to chlorosis compared with WT plants (Figure 3B). Furthermore, the OE seedlings exhibited significantly greater fresh weight, dry weight, root length, shoot length, and chlorophyll content under heat, salt, and drought stress compared with the WT plants (Figures 3C-E and Supplementary Figures 5A-C).

**FIGURE 4:**
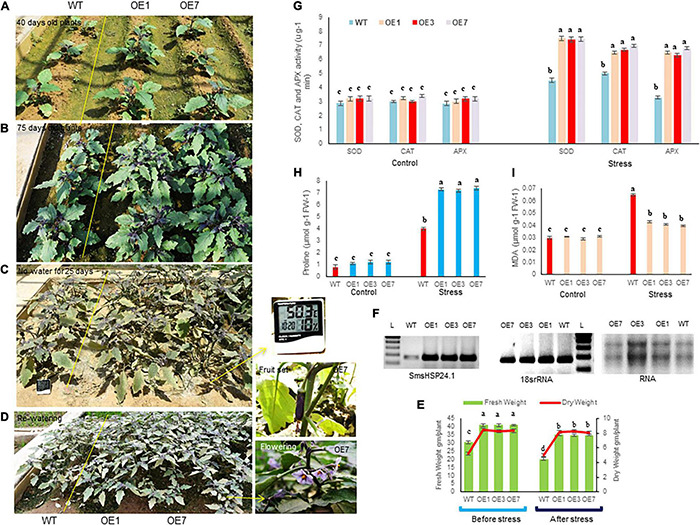
Transgenic plants overexpressing *SmsHSP24.1* maintained physiological balance at the field level under combined (heat and drought) stress. **(A,B)** Mature transgenic as well as WT plants grown in normal field conditions. **(C)** Phenotypes of 75 days old eggplant seedlings imposed by water withdrawal for 25 days with 45°C average heat from nature. **(D)** Phenotypes of transgenic and WT seedlings after re-watering for 15 days. Initiation of flower and fruit sets after recovery from stresses. **(E)** Total fresh weight and dry weight were significantly higher in all transgenic seedlings under control and stress conditions. Data are M ± SD (*n* = 3). The letters a, b, c, and others indicate significant differences between different lines (*P* < 0.05). **(F)** Semi-quantitative RT-PCR analysis of *SmsHSP24.1* from field-grown OE and WT lines show increased accumulation of SmsHSP24.1 transcript in all OE lines both under control and stress conditions. **(G–I)** Different biochemical parameters such as proline, MDA, and ROS-scavenging enzymes such as superoxide dismutase (SOD), ascorbate peroxidase (APX), and catalase (CAT) were determined in transgenic as well as WT lines. Data are *M* ± *SD* (*n* = 3). The letters a, b, c, and others indicate significant differences between samples from different OE lines (*P* < 0.05).

A field study of combined heat and drought stress was also conducted at the ICGEB net house facility, New Delhi. In here, two and half-month-old seedlings of both OE lines and WT plants were subjected to 25 days of water withdrawal and an average of 43°C air and 50°C soil temperature at the reproductive stage. Under such conditions, most WT seedlings were found to be severely injured, whereas OE lines remained green and healthy (Figures 4A-C). OE lines (OE1, OE3, and OE7) recovered quickly without any effect on flowering and fruit setting after withdrawal of stress, whereas the WT plants were completely retarded (Figure 4D). Physiological parameters also showed higher fresh and dry weight in all OE lines compared with WT under control and stress conditions (Figure 4E). Besides, semi-quantitative RT-PCR analysis showed increased accumulation of *SmsHSP24.1* transcript in all OE lines both in control and combined stressed plots compared with WT (Figure 4F). Enzymatic activity of ROS-scavenging enzymes, such as SOD, APX, and CAT was also monitored in both OE and WT lines from the combined stress plot. OE lines were found to maintain a high level of all the ROS-scavenging enzymes compared with WT plants (Figure 4G). Similarly, proline content showed an increase in overexpressed lines, whereas MDA content had decreased significantly after stress treatment in the OE lines compared with WT plants (Figures 4H,I). This data was found to correlate with RNA-seq, and semi-quantitative RT-PCR analysis carried out on 3-week-old heat stress OE lines.

### SmsHSP24.1 Overexpressing Plant Maintains an Elevated Level of Cellular Reactive Oxygen Species Under Both Normal and Stress Conditions

**FIGURE 5:**
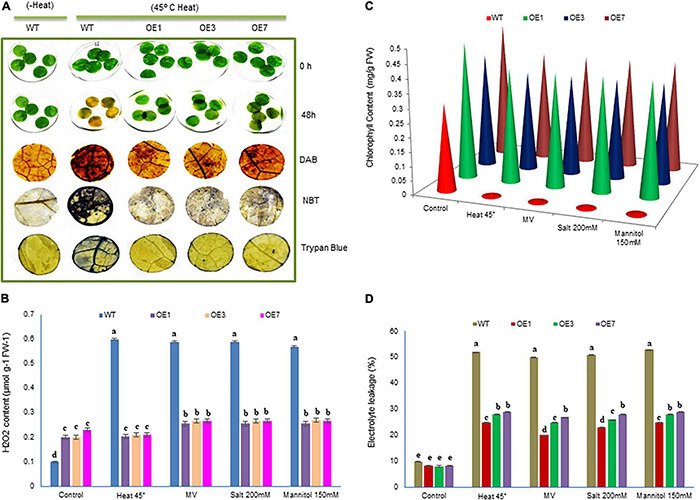
Analysis of cellular damage in response to heat, salinity, and drought stresses. **(A)** Leaf strips of WT and three transgenic eggplants lines (OE) under 45°C heat stress for 48 h. Untreated (without heat) WT leaves were used as a control. Relative H_2_O_2_ and O^2–^ accumulation and dead cells in the leaf strips of transgenic and WT lines were detected by histochemical staining. **(B–D)** H_2_O_2,_ chlorophyll content, and electrolytic leakage were measured in response to heat, salinity, MV (methyl viologen), and osmotic stresses. Data are *M* ± *SD* (*n* = 3). The letters a, b, c, and others indicate significant differences between different lines (*P* < 0.05).

To evaluate the impact of overexpressed *SmsHSP24.1* in mitigating ROS damage, we conducted leaf disc assay separately for heat (45°C heat for 36 h), osmotic stress (150 mM Mannitol), salt (200 mM NaCl), and MV (10 μM) to investigate the H_2_O_2_, O^2–^ contents and cell death after stress (Figure 5 and Supplementary Figure 6). Reduced and slow chlorosis was observed for SmsHSP24.1 overexpressing event compared with WT plants after stress treatments. The OE lines overexpressing *SmsHSP24.1* showed a significantly lower level of ROS production and cell death compared with WT plants after MV (Supplementary Figure 6A) and heat treatment as were evident by DAB, NBT, and Trypan blue staining (Figure 5A). We also noticed that the H_2_O_2_ levels in WT plants were approximately 3- to 4-fold higher than OE lines after the heat, salt, mannitol, and MV treatment (Figure 5B). However, it was interesting to note that under normal conditions, the OE lines maintained a slightly higher cellular H_2_O_2_ level compared with WT plants. However, we have observed that this slight increment of cellular ROS in OE lines did not hamper the growth under normal environmental conditions, while no lethal increment in the level of H_2_O_2_ was observed in OE lines compared with WT plants upon stress treatment (Figure 5B). This finding was also supported by a comparative analysis of chlorophyll to be retained and ion leakage between OE lines and WT plants. OE lines were found to prevent chlorophyll damaged and reduced ion leakage probably due to the protection from oxidative damage through decreased ROS production under stress conditions (Figures 5C,D). As described in the previous data (Figure 4C), OE lines are able to maintain a higher level of ROS scavenging enzymes compared with WT plants due to which ROS production could not be increased up to lethal level.

### SmsHSP24.1 Overexpressed Lines Exhibit Early Seed Germination and Seedling Vigor

We observed a significant rate of early seed germination and seedling vigor, both *in vitro* and field condition in OE lines (OE1, OE3, and OE7) compared with WT (Supplementary Figure 7A). Furthermore, a 95% germination rate was observed within 3 to 4 days in OE lines (OE1, OE3, and OE7) in the growth medium, while 6 days were required to attain an 80% germination rate in WT (Supplementary Figure 7A). A similar result was observed when seeds were sown in the field under normal conditions. Physiological parameters obtained at 2 weeks after germination revealed significantly higher total fresh weight, dry weight, root length, shoot length, and chlorophyll content in all OE seedlings compared with WT seedlings (Supplementary Figures 7B-D).

**FIGURE 6:**
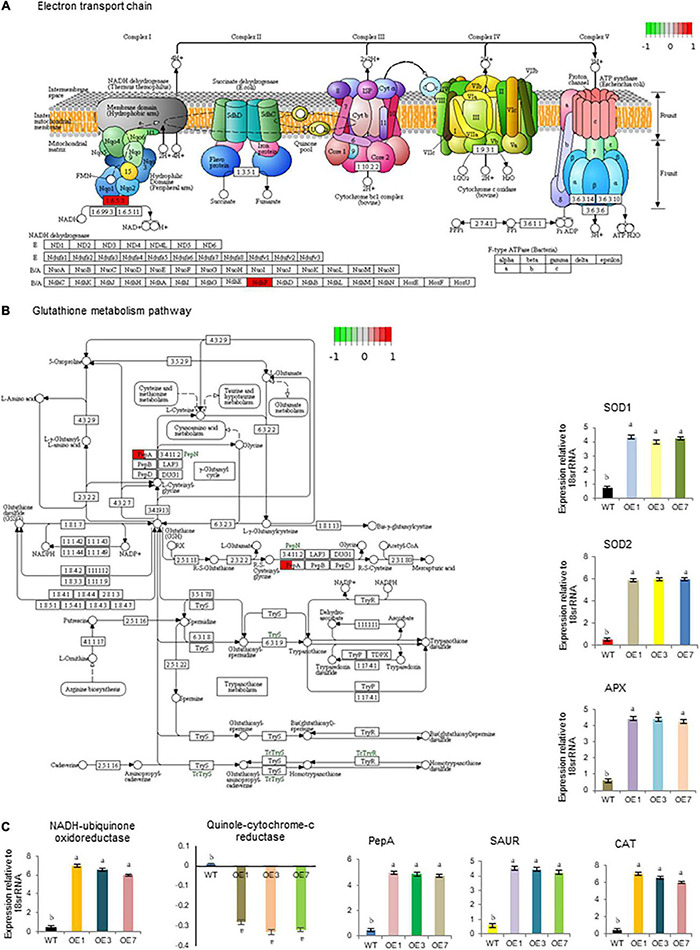
Important biological pathways significantly affected SmHSP24.1 overexpressed transgenic (OE) plants as compared with wild-type (WT). **(A)** First protein complex NADH-ubiquinone oxidoreductase significantly affected the electron transport chain (ETC). **(B)** PepA (aminopeptidase) positively affected the Glutathione metabolism pathway. **(C)** Time-dependent relative quantitative RT-PCR validation of some selected genes (*NADH-ubiquinone oxidoreductase*, *Quinol-cytochrome c- oxidoreductase*, *PepA*, *SOD1*, *SOD2*, *CAT*, *APX*, and *SAUR*) highly affected in these pathways. mRNA level was normalized with respect to housekeeping gene *18srRNA* and fold change in expression was calculated as compared with WT sample. RT-qPCR data are *M* ± *SD* (*n* = 3). The letters a, b, c, and others indicate significant differences between different lines (*P* < 0.05).

We further conducted transcriptome analysis to understand the possible molecular mechanisms underlying the effect of early seed germination and seedling vigor. The comprehensive transcriptome data have been presented separately; however, changes in transcription were noticed in three key regulatory pathways; ETC and GSH, as well as auxin biosynthesis and transport (Figures 6A,B and Supplementary Figure 8). In ETC, *NADH-ubiquinone oxidoreductase* transcript was found to be significantly upregulated while *quinol-cytochrome c- oxidoreductase* was slightly down-regulated in OE lines compared with WT lines under both normal and stressed conditions. qPCR cross-validation also revealed similar results (Figure 6C). *PepA* (*aminopeptidase*) gene from the GSH pathway was found to be highly upregulated in OE lines. It was approximately 5-fold higher in OE lines compared with WT (Figure 6C). Similarly, significant upregulation was also observed for auxin carrier proteins like auxin-responsive *SAUR-lik*e genes. Likewise, RNA-seq values of differentially expressed transcripts of *SOD1*, *SOD2*, *CAT*, and *APX* in OE lines, further confirmed by qPCR (Figure 6C).

### SmsHSP24.1 Overexpression Leads to Global Transcriptomic Reprogramming

Transcriptome profiling of OE lines compared with WT plants further provide evidence of a significant impact on global transcriptome due to the overexpression of *SmsHSP24.1.* During this experiment, an average of 56.1 and 83.5 million raw reads (2 × 100 bp) were generated from transgenic (OERT) and WT (WTRT) lines, respectively, under controlled environmental conditions. Similarly, an average of 73.5 and 82.5 million raw reads (2 × 100 bp) were generated under the heat-stressed conditions at 45°C for 2 h from transgenic (OE2hH) and WT (WT2hH) line respectively.

**FIGURE 7:**
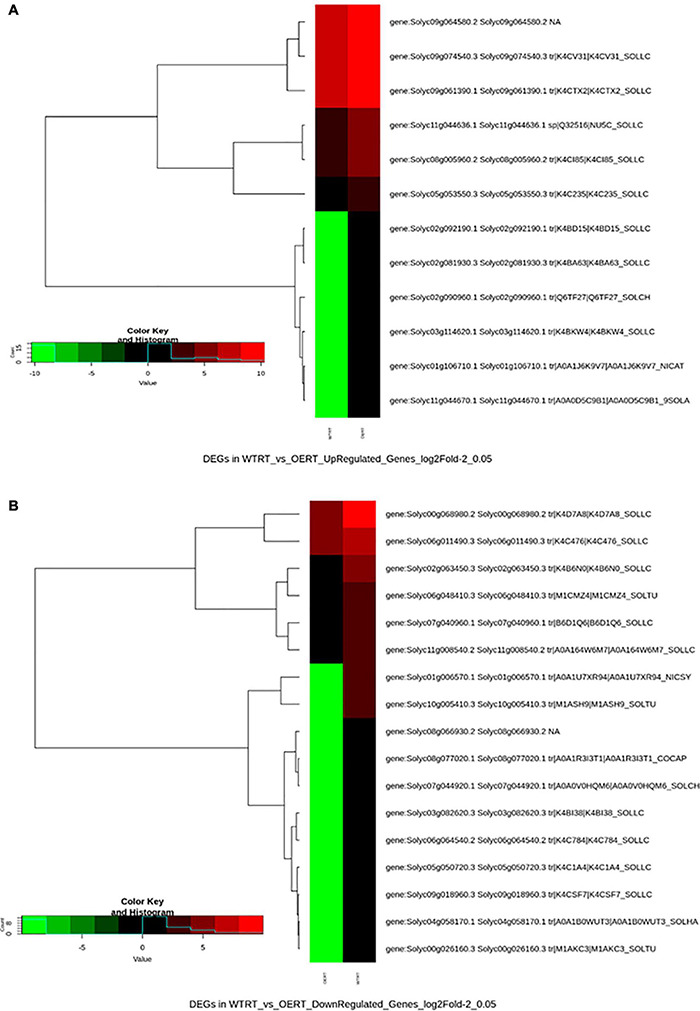
Heat map of differentially expressed genes (DEGs) in SmHSP24.1 overexpressed transgenic (OE) and wild type (WT) lines under control conditions**. (A)** Heatmap generated from differentially expressed upregulated genes in OERT vs. WTRT lines. **(B)** Heatmap generated from differentially expressed down-regulated genes in OERT vs. WTRT lines.

**FIGURE 8:**
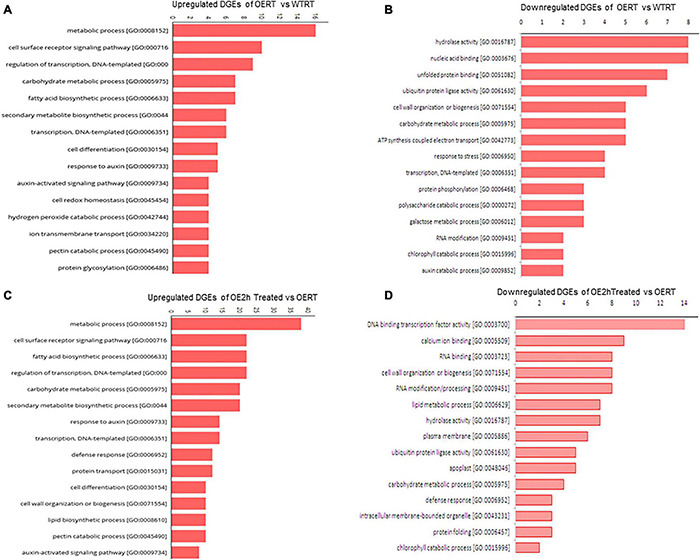
Gene ontology (GO) classification of DEGs in SmHSP24.1 overexpressed OE and WT lines under control and stress conditions. **(A,B)** GO for both up- and down-regulated differentially expressed genes in SmHSP24.1 overexpressed OERT vs. WTRT lines under control environmental growth condition. **(C,D)** GO for both up- and down-regulated differentially expressed genes in SmHSP24.1overexpressed transgenic line under heat stress conditions. The y-axis and x-axis indicate the names of clusters and the gene number of each cluster respectively. Only the biological processes were used for GO term analysis. OERT: SmHSP24.1overexpressed line in room temperature, WTRT: WT line in room temperature, OE2h: SmHSP24.1overexpressed line under 2 h heat (45°C) stress condition.

After adaptor trimming and base quality check, it was found that more than 95% of total reads were of high quality (HQ) except for the WT2hH plant, in which after the removal of rRNA, the value was 87%. Mapping of cleaned reads with tomato reference genome and gene model downloaded from Sol Genomics Network (Fernandez-pozo et al., 2015) found 65.6% of OERT, 52.8% of WTRT, 56.88% of OE2hH, and 57.76% of WT2hH reads to be mapped (Supplementary Table 3). Differential gene expression analysis revealed significant transcription alteration in some of the physiologically important pathways (Figures 6A,B and Table 1). Heat map illustration with cluster dendrogram based on FPKM plot and GO classification of DEGs in WT and OE also revealed the up- and down-expression of important genes (Figures 7, 8 and Supplementary Figures 9–11). Among the several DEGs, *Photosystem II reaction center protein M* (*Solyc09g064580*), *Chloroplastic matK* (*Solyc09g061390*), genes involved in electron transport chain (*Solyc09g074540*, *Solyc11g044636*) were significantly upregulated in OE_vs_WT samples at RT (Figure 7 and Table 1), while *SOD* (*Solyc01g067740*), ABC transporter that regulates lipid transporter (*Solyc03g113070*), *GLB1*(nitrogen regulatory P-II-like protein) regulated fatty acid biosynthesis (*Solyc07g008240*) (Duan et al., 2017), in OE_vs._WT samples after heat treatment, respectively (Supplementary Figure 9). Similarly, significant down-regulation was also observed in transmembrane transporters like *F-box domain-containing protein* (*Solyc07g044920*), *iron-nicotianamine transporter* (*Solyc03g082620*), *calcium-binding protein CML44* (*Solyc04g058170*), and *LOB1* (*Solyc06g064540*) under normal conditions, while *ADF-H actin-binding protein* (*Solyc02g063450*), cell division cycle protein- *CDC27B* (*Solyc03g061590*), cell number control protein- *PLAC* (*Solyc02g079390*), and FA_desaturase domain-containing protein (*Solyc10g011810*) in WT eggplants compared with OE lines upon heat treatment (Supplementary Figure 10 and Supplementary Table 1). GO term-based gene classification for DEGs in WT, and OE samples also revealed a large number of genes affected significantly. Twelve major pathway-related genes were found to have distinctive differential expression in OE treated lines compared with WT (Figure 8 and Table 1). Similarly, a total of 185 genes are down-regulated in the same event (Figure 8 and Supplementary Table 1). Among the genes for transcription factors, BZIP transcription factor (*Solyc01g109880.3*), *ethylene-responsive factor 2*; ethylene-binding protein (*Solyc09g075420.3*), and NAC4 domain protein (*Solyc11g017470.2*) were found to be down-regulated whereas a total of 18 different transcription factors (Tfs), most of which were auxin-responsive/ethylene-responsive, DREB, GATA, and TCP family transcripts were found to be upregulated in OE lines.

**TABLE 1:** Upregulated DEGs in overexpressed OE lines compared with the wild-type (WT) eggplant line.

**Gene symbol**	**log2(fold change)**	**Protein name**
**DGEs related to auxin-response and transport**		
Solyc01g110900.1	2.22	auxin-induced protein 15A-like
Solyc01g110940.3	2.07	Auxin-induced protein 15A; auxin-induced protein 15A-like
Solyc03g033590.1	2.59	Auxin-induced SAUR-like protein; auxin-induced protein 15A
Solyc03g082510.1	2.21	Auxin-responsive protein SAUR32
Solyc03g123410.1	4.40	auxin-binding protein ABP19a-like
Solyc05g008060.3	2.26	Auxin efflux carrier component
Solyc06g053840.3	4.58	Auxin-responsive protein
Solyc07g041720.1	3.24	Auxin-binding protein ABP19a; auxin-binding protein ABP19a-like
Solyc09g007810.3	3.36	Auxin response factor
Solyc09g083280.3	2.02	Auxin-responsive protein
Solyc10g080880.2	2.39	Auxin efflux carrier component
Solyc11g069190.2	2.31	Auxin response factor
Solyc11g011660.2	2.73	Uncharacterized protein
Solyc06g053260.1	3.18	Small auxin-up protein 58
**DGEs related to cell wall reorganization**		
Solyc02g087060.3	2.28	WAT1-related protein
Solyc02g091920.3	4.04	Xyloglucan endotransglucosylase/hydrolase
Solyc02g092840.1	3.44	xyloglucan galactosyltransferase XLT2-like
Solyc03g071570.3	2.73	Pectate lyase
Solyc05g014000.3	3.49	Pectate lyase
Solyc05g055840.3	2.64	Putative UDP-glycosyltransferase 86A1-like
Solyc05g055845.1	2.47	Putative UDP-glycosyltransferase 86A1-like
Solyc06g009190.3	2.59	Pectinesterase
Solyc06g062580.3	2.07	Beta-galactosidase
Solyc06g083580.3	2.49	Pectate lyase
Solyc07g017600.3	3.14	Pectinesterase
Solyc07g049610.1	3.23	xyloglucan galactosyltransferase XLT2-like
Solyc07g052690.3	2.38	Beta-amylase
Solyc07g052695.1	2.46	Beta-amylase
Solyc07g052980.3	2.64	Xyloglucan endotransglucosylase/hydrolase
Solyc08g075020.3	3.49	Pectin acetylesterase
Solyc08g079040.1	2.14	Putative xyloglucan galactosyltransferase gt19
Solyc09g008320.3	2.08	Xyloglucan endotransglucosylase/hydrolase
Solyc09g091430.3	4.28	Pectate lyase
Solyc11g005770.2	3.38	Pectinesterase
Solyc02g063140.3	3.92	3-ketoacyl-CoA synthase
**DGEs related to lipid biosynthesis**		
Solyc02g069490.3	2.52	Sterol side chain reductase
Solyc02g085870.3	3.24	3-ketoacyl-CoA synthase
Solyc03g025320.3	4.43	Alcohol acyl transferase
Solyc04g009380.2	2.41	S-acyltransferase; Palmitoyltransferase
Solyc05g009270.3	3.88	3-ketoacyl-CoA synthase
Solyc05g012790.3	2.68	S-acyltransferase; Palmitoyltransferase
Solyc05g013207.1	3.79	3-ketoacyl-CoA synthase
Solyc06g074390.3	2.88	Fatty acyl-CoA reductase
Solyc08g067260.3	2.26	3-ketoacyl-CoA synthase
Solyc09g083050.3	3.07	3-ketoacyl-CoA synthase
Solyc10g011820.3	2.55	Putative delta (8)-fatty-acid desaturase-like
Solyc11g072990.2	2.50	3-ketoacyl-CoA synthase
Solyc12g006820.2	2.23	3-ketoacyl-CoA synthase
**DGEs related to cellular redox homeostatsis**		
Solyc01g006290.3	3.63	Peroxidase
Solyc01g006300.3	3.19	Peroxidase
Solyc02g087190.1	2.12	Peroxidase
Solyc03g093180.1	3.24	Peroxisomal membrane protein 11-4
Solyc02g087850.1	2.30	Putative ovule protein
Solyc05g053300.3	2.37	Dihydrolipoyl dehydrogenase
Solyc08g062970.1	2.48	Putative glutaredoxin-C6-like
Solyc08g082590.3	2.01	Uncharacterized protein
Solyc10g006970.3	2.20	Uncharacterized protein
Solyc10g007110.3	2.26	Tyrosine aminotransferase 1
Solyc11g066390.2	3.93	Superoxide dismutase [Cu-Zn]
**DGEs related to histone modification**		
Solyc01g110150.2	2.04	Putative histone-lysine N-methyltransferase SETD1B-like
Solyc02g077480.1	2.63	Histone H3.2; Histone H3.1
Solyc06g005420.1	2.08	Histone H4; CaH4
Solyc06g084020.3	3.64	Histone H1
Solyc10g008910.1	2.80	Histone H3.2; Histone H3.1
Solyc11g073250.2	2.55	Histone H2A
**DGEs related to chlorophyll biosynthetic process**		
Solyc01g105030.3	2.19	Chlorophyll a-b binding protein CP24 10A, chloroplastic; CAB-10A; LHCP
Solyc01g105050.3	2.51	Chlorophyll a-b binding protein CP24 10B, chloroplastic; CAB-10B; LHCP
Solyc06g063360.3	2.10	Chlorophyll a-b binding protein, chloroplastic
Solyc10g006230.3	2.06	Chlorophyll a-b binding protein 7, chloroplastic; LHCI type II CAB-7
Solyc10g018580.1	2.27	Protein TIC 214; Translocon at the inner envelope membrane of chloroplasts 214
Solyc11g021290.2	2.03	Protein TIC 214; Translocon at the inner envelope membrane of chloroplasts 214
Solyc11g021300.1	2.41	Protein TIC 214; Translocon at the inner envelope membrane of chloroplasts 214; AtTIC214
Solyc12g035550.1	2.95	Protein TIC 214; Translocon at the inner envelope membrane of chloroplasts 214; AtTIC214
Solyc10g018300.2	2.22	Transketolase, chloroplastic; TK
Solyc01g094750.3	4.14	cytochrome P450 86A8-like
Solyc01g107730.3	3.30	CycD3
Solyc02g089160.3	2.47	Cytochrome P450 85A1; C6-oxidase; Dwarf protein 1.10.2.2
Solyc03g111950.3	3.27	cytochrome P450 71A3-like
Solyc04g054260.3	2.32	cytochrome P450 CYP736A12-like
Solyc05g055400.3	5.046	CYP77A19
Solyc08g081220.1	2.68	cytochrome P450 86A22
Solyc11g007540.2	2.67	CYP77A20
Solyc09g064500.3	2.26	Photosystem II reaction center Psb28 protein
Solyc06g060340.3	2.66	Photosystem II 22 kDa protein, chloroplastic; CP22
Solyc01g109260.3	2.44	Putative plastid division protein PDV2-like
**DGEs related to transcription factors**		
Solyc04g054910.3	3.07	ethylene-responsive transcription factor RAP2-13-like
Solyc05g052030.1	2.73	Ethylene response factor 4
Solyc08g008305.1	2.33	ethylene-responsive transcription factor ERF061
Solyc01g110310.3	3.56	GATA transcription factor
Solyc02g067340.3	2.15	Transcription factor
Solyc02g077710.1	3.87	GATA zinc finger domain-containing protein 14-like
Solyc03g121240.1	2.50	transcription factor bHLH87 isoform X2
Solyc06g070900.3	3.21	TCP transcription factor 17
Solyc10g055410.2	3.29	Transcription factor
**DGEs related to ATP synthesis coupled electron transport**		
Solyc01g017110.1	3.73	NAD(P)H-quinone oxidoreductase subunit 5, chloroplastic; NADH-plastoquinone oxidoreductase subunit 5
Solyc01g017333.1	4.48	Uncharacterized protein
Solyc01g065620.1	2.10	NAD(P)H-quinone oxidoreductase chain 4, chloroplastic; NAD(P)H dehydrogenase, chain 4; NADH-plastoquinone oxidoreductase chain 4
Solyc03g043610.2	3.17	ATP synthase subunit a; F-ATPase protein 6
Solyc11g044636.1	2.12	NAD(P)H-quinone oxidoreductase subunit 5, chloroplastic; NAD(P)H dehydrogenase subunit 5; NADH-plastoquinone oxidoreductase subunit 5.

Well-known ROS-scavenging genes such as *SOD*, *catalase*, *ascorbate*-*peroxidase* were significantly upregulated in OE lines under both normal and treated conditions. An extensive set of genes involved in fatty acid biosynthesis (28 in number, Table 1), chlorophyll biosynthesis, genes involved in the developmental pathway (32 in number), and mitochondrial integral membrane protein have identified upregulated. Among phytohormones, auxin biosynthesis/carrier-related transcripts upregulated in OE lines (Table 1). A total of 14 auxin pathway genes were significantly upregulated in OE lines compared with WT lines. Transcript for auxin-induced *SAUR-like* protein showed 4.3-fold upregulation. Significant upregulation was also observed for cytokinin oxidoreductase and cytokine-responsive transcripts in OE lines. This considerable alteration in transcription might have significant effects on OE lines, and our RNA-Seq data reveal substantial transcriptional reprogramming in several physiologically essential pathways of the OE lines (Table 1). DEGs involved in other essential pathways were also found to be down-regulated (Supplementary Table 1), such as 8 DEGs involved in hydrolase activity [GO:0016787], 6 DEGs with ubiquitin-protein ligase activity [GO:0061630], 5 DEGs of cell wall organization or biogenesis pathway [GO:0071554] and 2 each of auxin catabolic process [GO:0009852] and chlorophyll catabolic process [GO:0015996].

## Discussion

Unlike other organisms, plants have diverse and abundant low-molecular-weight (12-42 kD) sHSP (Sun et al., 2016; Waters and Vierling, 2020). However, until its chaperone activity, the other roles of organelle targeted sHSPs have not been well studied. It is worth mentioning that Mito- and ER-localized sHSPs have evolved explicitly in plant lineage and are not found in other eukaryotes (Waters and Vierling, 2020) except for the presence of HSP22 in *Drosophila*. Interestingly, it is now well documented that apart from the chaperone activity, HSP22 has been essential for the longevity of *Drosophila* (Morrow and Tanguay, 2015). Therefore, it was crucial to understanding the evolutionary advantage that a plant acquires from the Mitochondria-targeted sHSPs.

We had identified and characterized a novel sHSP “SmsHSP24.1” localized in the mitochondria of eggplants. *In silico* analysis revealed the presence of a conserved mitochondrial signal aligned with the previously reported Mito-sHSP from different plants. Agroinfiltration on tobacco-leaf and eggplant cell suspension culture using MitoTracker^TM^ FM conclusively proved that this novel SmsHSP24.1 is mitochondria localized protein. We used eggplant cell suspension to determine host protein localization. According to an earlier report, phylogenetic distances are expected to have an impact on protein trafficking and localization in different host systems (Kirienko et al., 2012). Also, *in silico* prediction of only one copy of *Mito-sHSP* (*SmsHSP24.1*) in the eggplant genome was confirmed by Southern blot analysis (Supplementary Figure 4F). Therefore, we further studied to understand the role of SmsHSP24.1 under both normal and stressed conditions.

Expression analysis in WT plants clearly showed that *SmsHSP24.1* is sensitive to different environmental stresses, especially to heat, salt, and mannitol stresses. A rapid increase of its transcript level indicates an early response of *SmsHSP24.1* against stress conditions. This is important in the context of its localization. The mitochondrion is the powerhouse of the cell and cell metabolism (Morrow and Tanguay, 2015) and also a source of cellular ROS production during stress (Huang et al., 2016). Thus, maintenance of mitochondrial protein homeostasis was crucial for survival. We had discussed the possible role of SmsHSP24.1 in the later part of the discussion. To further understand its physiological role, we developed overexpressing eggplant lines and have studied the molecular activity of SmsHSP24.1 both *in vitro* and *in vivo*.

Unlike other chaperons, the affinity of sHSPs to selectively bind with aggregation-prone proteins in an ATP-independent way is high (Kuang et al., 2017). A thermal survivability test revealed the ability of SmHSP24.1 protein protection against aggregation of *E. coli* cells up to 55°C for 1 h. Our results complement similar findings from previously reported thermotolerance of RcHSP17.8 in *E. coli* (Jiang et al., 2009); CsHSP17.2 in *E. coli* and *Pichia pastoris* cells (Zhang et al., 2015; Wang et al., 2017). It was conclusively understood from our study that *E. coli* cells were able to survive under heat stress due to the heterologous overexpression of SmsHSP24.1. The mechanisms by which SmsHSP24.1 performs the chaperoning activity are difficult to define due to the limited structural information (Rutsdottir et al., 2017). Our Electron microscopy study suggested the formation of the polyhedral structure by SmsHSP24.1 which could bind with its client protein even at a higher temperature of 50°C. Our results which were in agreement with previous findings (Zhang et al., 2015) suggested a possible mechanism of ATP- independent chaperon activity of sHSPs induced by the formation of multimeric topological structures.

Apart from chaperon activity, seeds from OE lines showed a quicker germination rate compared with the WT plants, along with rapid seedling growth and vigor irrespective of the integration site. Altered phenotypes such as pollen growth and development, seed and root development, response to pathogen and UV damage, *etc.* have also been reported in previous studies of sHSPs (Sun et al., 2002; Murakami et al., 2004; Zou et al., 2009). Agrobacterium-mediated transformation can cause insertional inactivation of functional genes which can alter phenotype, but all our lines with different copy numbers show an even rate of germination. This indicates to be an effect of SmsHSP24.1 overexpression, opening up opportunities for exploiting this feature for the development of rapid germination, quick seedling growth, and vigor. Such traits are essential in commercial cultivation for high yield and efficient resource utilization (Finch-Savage and Bassel, 2016).

It has progressively emerged that cellular ROS homeostasis is crucial for seed germination and has evolved as an “oxidative window of seed germination” regulation (Bailly et al., 2008; El-Maarouf-Bouteau and Bailly, 2008). Mitochondria are the central regulators of ROS production (Noctor et al., 2007). It has been well accepted that NADH:ubiquinone oxidoreductase (complexes I) and ubiquinol:cytochrome c oxidoreductase (complexes III) are the main producers of superoxide production in higher eukaryotes (Dröse and Brandt, 2008; Larosa and Remacle, 2018). Our transcriptome data revealed interesting results that hypothesize the possible role of SmsHSP24.1 in rapid seed germination. The transcript level was lower for quinol-cytochrome c- oxidoreductase (EC 1.10.2.2), whereas it was higher for NADH-ubiquinone oxidoreductase (EC 1.6.5.3) in OE lines under both stressed and normal conditions compared with WT plants. This differential expression of electron acceptor and donor, especially for NADH-ubiquinone oxidoreductase, the first electron receptor, and quinol-cytochrome c- oxidoreductase, the third complex in the electron transport chain might have creates an imbalance in the electron gradient which during normal growth conditions leads to an elevated level of cellular ROS. Besides, biochemical analysis of ROS production also suggests a slightly higher ROS level in OE lines than WT plants under normal conditions. This supports our hypothesis that overexpression of SmsHSP24.1 increases germination by altering the cellular ROS gradient. In contrast to the elevated cellular ROS, no negative growth effect was observed in SmsHSP24.1 OE lines. The probable reason is the simultaneous elevation of several necessary ROS-scavenging transcripts as observed in RNA-Seq analysis, such as Mito-*superoxide dismutase* (*Solyc06g048410.3, Solyc06g048420.2*), *catalase* (*CAT*) (*Solyc04g082460.3*), *APX* (*Solyc09g007270.3*) in OE lines compared with WT. Some key cell redox homeostasis transcripts, e.g., *PepA glutaredoxin*, *proline dehydrogenase*, *glutamate synthase* genes have also been found to be upregulated in OE lines. qPCR expression crosscheck along with enzymatic analysis supports these findings. These indicate *SmsHSP24.1* overexpression leads to an elevation of cellular ROS at normal growth conditions without being lethal to the host cell; however, this caused a reprogramming of global transcripts in OE lines. Rapid seedling growth is determined by hypocotyl elongation occurring through cell expansion which is centrally controlled by the auxin hormone. Previous studies have demonstrated that overexpression of *AtSAUR36* (Stamm and Kumar, 2013), *AtSAUR41* (Kong et al., 2013), *AtSAUR19* (Spartz et al., 2012), and *AtSAUR63* (Chae et al., 2012) promote hypocotyl elongation as a result of increased cell expansion. Similar to these findings, our RNA-seq data suggest overexpression of a set of genes related to auxin-biosynthesis and carrier pathway. Overexpression of selected eggplant-specific auxin-responsive protein *SAUR32* gene was validated by their expression analyses in the OE lines. These findings suggested that OE lines maintain a significantly higher *SAUR32* transcript level and other auxin biosynthesis and carrier transcripts that positively regulate hypocotyl growth, hinting at a complex gene regulatory network of SmsHSP24.1 protein on plant growth and development.

Ribonucleic acid-seq data also provide insight into the molecular mechanism of increased heat tolerance of OE plants. Results suggest that cellular transcript reprogramming not only maintains redox homeostasis but also alters several key physiological pathways. Field analysis of OE lines under combined severe heat and drought conditions demonstrate better crop performance over WT eggplants. RNA-seq analyses also suggest altered cell wall biogenesis in OE lines (Table 1). It has been well documented through previous studies that the modification of the biophysical properties of a cell wall was a crucial response toward environmental stimuli such as heat, necessary to maintain the overall function and growth (Lima et al., 2013; Wu et al., 2018).

Other significant altered transcripts in our study were found to be related to lipid metabolism, chloroplast (including photosystem II receptor protein) and mitochondrial membrane proteins, histone and histone modification, and transcription factor genes. Profound transcriptional reprogramming during heat stress has been reported in several plants affecting several traits (Zou et al., 2011; Barah et al., 2013). Our results also showed a similar effect on the global transcriptome in OE lines. A dynamic change in lipid composition in the cell membrane is crucial during stress signaling and response (Hou et al., 2016). Among 13 DEGs, fatty acyl Co-A reductase (FAR), a key enzyme in the biosynthesis of long-chain fatty alcohols such as cuticular wax (Rowland et al., 2006), works as an interface between plants and their biotic and abiotic environments, significantly restricting non-stomatal water loss and serves as the first line of defense (Chai et al., 2018).

Similarly, a total of 20 chloroplast-related DEGs have been found in OE lines. Chlorophyll is one of the major targets of heat stress damage, crucial for plant growth and development. Significantly higher upregulation of these genes is vital for chlorophyll development and photosynthesis during normal and stress conditions. Chromatin re-modeling is another way of regulating expression. Histone modification and alteration of DNA methylation patterns are crucial ways to counter environmental changes. This is often coordinated with dynamic changes in stress-responsive genes (Kim et al., 2015). Similarly, Tfs are also involved in normal growth and development and abiotic stress responses. Tfs, such as ethylene-responsive elements, have also been observed. Multiple transcriptions reprogramming due to an overexpression of SmsHSP24.1 confers abiotic stress tolerance and improved growth and development under normal environmental conditions.

**FIGURE 9:**
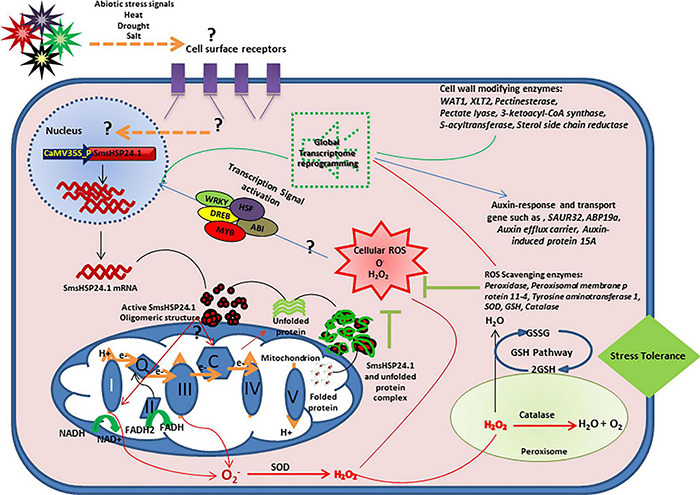
Hypothetical model for understanding the molecular mechanism of SmsHSP24.1-mediated plant abiotic stress response. Overexpression of SmsHSP24.1 might interact with mitochondrial electron transport chain (ETC) which leads to increased ROS. A set of reactive oxygen species (ROS) scavenging enzymes of the glutathione (GHS) pathway also upregulated and maintains ROS below lethal dose which might act as a transcriptional activation signal of several stress-induced transcription factors and ultimately lead to change in global transcription.

Finally, we had identified a novel mitochondrion localized SmsHSP24.1 protein in eggplant and characterized the same under both normal and stressed conditions. While overexpression of *SmsHSP24.1* enhanced abiotic stress tolerance, especially in temperatures up to 45°C in the field condition, rapid germination and seedling vigor were also observed. Thus, based on our findings, we had proposed a hypothesis for the possible mechanism of overexpression of SmsHSP24.1 and its impacts on ROS regulation and reprogramming of the global transcriptome in transgenic eggplant lines compared with WT (Figure 9). Further, it is interesting to study the targeted cytosolic or mitochondrial protein profiling in both normal and stress conditions. A recent study by Escobar et al. (2021), has shown that Mito-sHSP regulates cellular homeostasis by coordinating between different subcellular compartments especially between plastids, cytosol, and mitochondrion in *Arabidopsis*. Thus, in the future lines of work, comparative studies of protein profiling in OE lines against WT plants corresponding to the finding of our transcriptome analysis are useful to understand the regulatory mechanism of Mitochondria localized small HSPs in plant growth and development. Findings from the present study also suggested that mitochondria-targeted sHSPs provided an evolutionary advantage to plants that could be harnessed for the development of stress-resilient crop plants.

## Data Availability Statement

The original contributions presented in the study are publicly available. This data can be found here: National Center for Biotechnology Information (NCBI) BioProject database under accession number PRJNA750594.

## Author Contributions

MR, MS, IA, BB, and MK designed the experiments. MK, BB, and VP performed experiments and data analysis. MR and MS wrote the manuscript. MR, MS, IA, HK, SH, and CK supervised the project and revised the manuscript. All authors contributed to the article and approved the submitted version.

## Conflict of Interest

The authors declare that the research was conducted in the absence of any commercial or financial relationships that could be construed as a potential conflict of interest.

## Publisher’s Note

All claims expressed in this article are solely those of the authors and do not necessarily represent those of their affiliated organizations, or those of the publisher, the editors and the reviewers. Any product that may be evaluated in this article, or claim that may be made by its manufacturer, is not guaranteed or endorsed by the publisher.

## Footnotes

1
https://www.thermofisher.com

2
http://www.neb.com

3
http://www.cbs.dtu.dk/services/TargetP/

4
https://www.qiagen.com

5
https://ftp.solgenomics.net/tomato_genome/Heinz1706/wgs/assembly/build_3.00/S_lycopersicum_chromosomes.3.00.fa.tar.gz
