# Supplementary material for: Mitochondria-Targeted SmsHSP24.1 Overexpression Stimulates Early Seedling Vigor and Stress Tolerance by Multi-Pathway Transcriptome-Reprogramming
Source: Front Plant Sci. 2021 Nov 23;12:741898. doi: 10.3389/fpls.2021.741898 (PMC8649800; doi:10.3389/fpls.2021.741898)
Supplement: Supplementary file 1 [file Data_Sheet_1.PDF]

A

```
                                                                                                       100
atccttatccagcaaaaagaatacaacgtgaagctcagtcaatcggcaagttcatacagctgcatctttctcttcagactcagaagcttgtaagcaatag
                                                                                                       200
cacaaATGGCAACTTCACTTGCTCTCAGGAGGGCTACTGCCTCATCGCTCTTCAATAGGCTCGTCAACCCTGTTCGCTCTGCCTCTGTTTTCCGATCCTT
      M  A  T  S  L  A  L  R  R  A  T  A  S  S  L  F  N  R  L  V  N  P  V  R  S  A  S  V  F  R  S  F
                                                                                                       300
CAATACTAACACTCAGATGACAGCTTATGACCAGGATGATCGTGATGTTGATGTCGAACGCCGGCCCGGTCGCTCCGTCTCTCGCCGCCGGGATGCTTTC
  N  T  N  T  Q  M  T  A  Y  D  Q  D  D  R  D  V  D  V  E  R  R  P  G  R  S  V  S  R  R  R  D  A  F
                                                                                                       400
CCTAGTTTGTTTTCAGATGTATTTGATCCATTCTCACCACCAAGGAGCGTGAGCCAGCTACTAAACATAATGGACCAAATGATGGATTCTCCATTTGTAG
 P  S  L  F  S  D  V  F  D  P  F  S  P  P  R  S  V  S  Q  L  L  N  I  M  D  Q  M  M  D  S  P  F  V
                                                                                                       500
CAGCACCGCGTTCTATGGGCACTGGAGTTGGTGCAAGAAGAGGATGGGACGTGAGAGAGGATGACAATGCTCTGTATATAAAAATGGATATGCCTGGGCT
A  A  P  R  S  M  G  T  G  V  G  A  R  R  G  W  D  V  R  E  D  D  N  A  L  Y  I  K  M  D  M  P  G  L
                                                                                                       600
CGACAAGGAGAACGTGAAGGTTGCGGTGGAGGAGAACACGTTGATCATCAAAGGAGAAGGTGAGAAAGAATCAGAGGATGAAGAGTATAGGAGGAGGTAC
  D  K  E  N  V  K  V  A  V  E  E  N  T  L  I  I  K  G  E  G  E  K  E  S  E  D  E  E  Y  R  R  R  Y
                                                                                                       700
TCGACGAGACTGGAAATTCCTCAAAATATGTACAAATTGGATGGAATTAAGGCGGAGATGAAGAATGGAGTGCTGAAAGTGGCTGTTCCAAAGGTGAAGC
 S  T  R  L  E  I  P  Q  N  M  Y  K  L  D  G  I  K  A  E  M  K  N  G  V  L  K  V  A  V  P  K  V  K
                                                                                                       800
AAGAAGAAAGGAAAGATGTTTTCAATGTTCAAATTGAGTGAgcttctagctgtgctaagtttgtactgtttgggtccgatgttataatttccccctttt
Q  E  E  R  K  D  V  F  N  V  Q  I  E  *
                                                                                                       900
tcttccccttctccgagctaggttggtctgcagtgatttgtctttgaaaaataaatgaaaatatggttgtaagggtctgttcatgtagctaactgggaag
                                                                                                      1000
ttgttgaatatactgttcttcaagtttttgttactacgagctttggacctgttatagattccaacttctgttgtgtaatggttatatggttctttctatt
tttcgatagaacattcatttttatattagaa
```

B

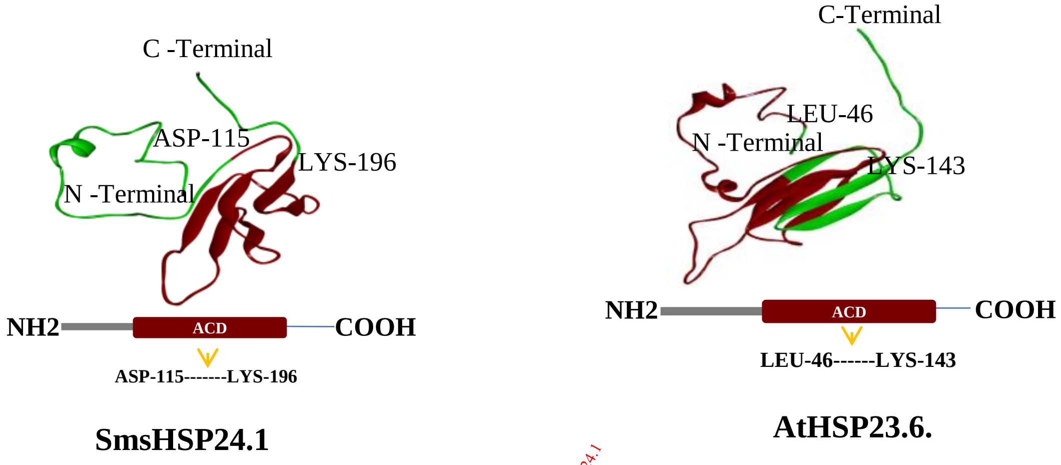

C

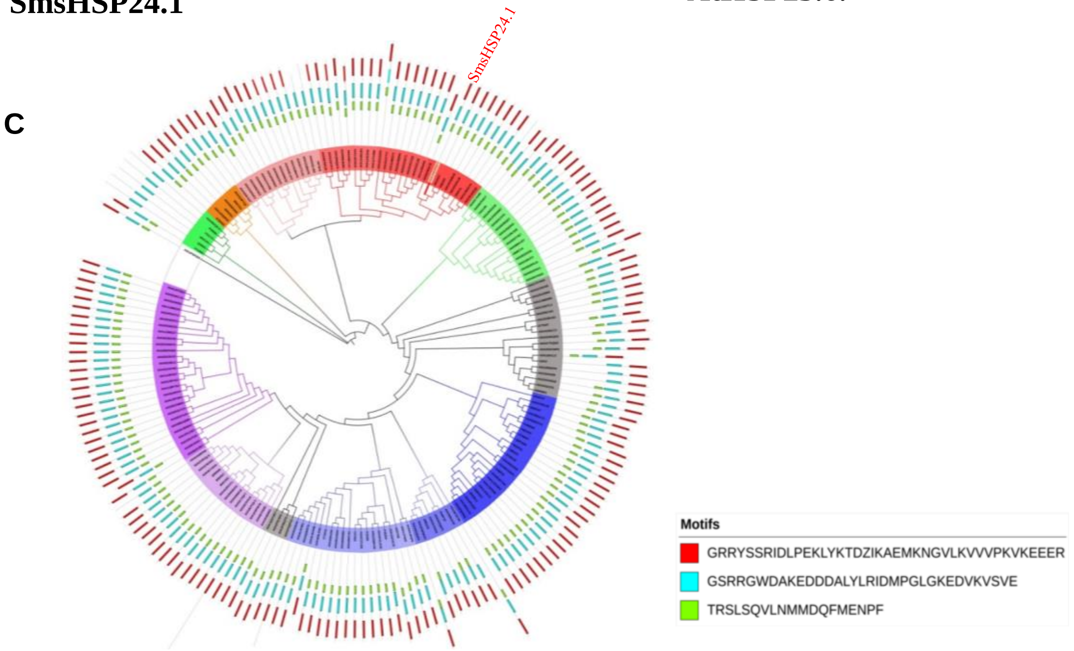

Figure S1. ***In silico*** **gene prediction, homology modelling and phylogenetic analysis of SmsHSP24.1 protein.**

(A) Eggplant full length putative mitochondrial small heat shock protein (1032 bp) gene. (B) Homology modelling of SmsHSP24.1 protein compared to AtHSP23.6. (C) Phylogenetic tree depicts diversity of mitochondria and chlorophyll-targeted small HSPs from diverse plant species

A

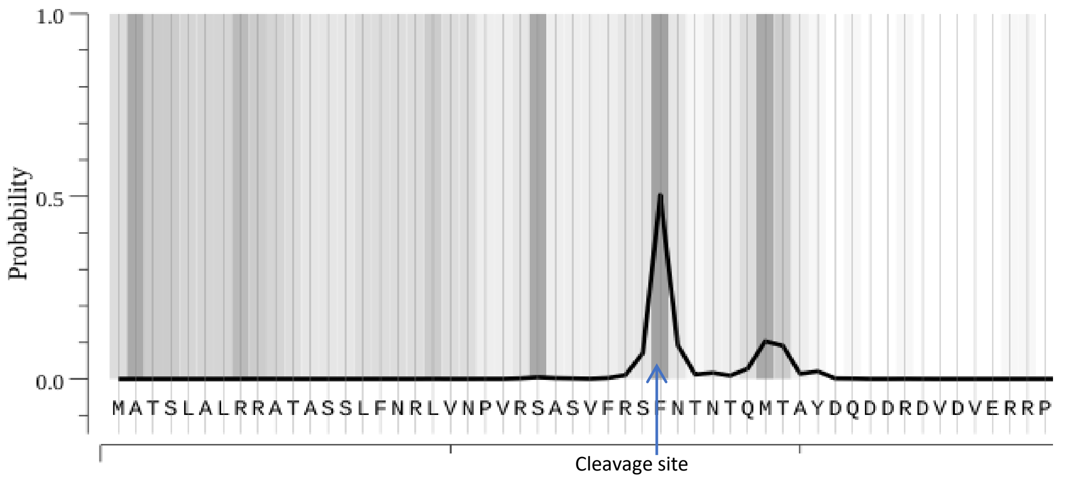

TargetP prediction	
Len	211
cTP	0.221
**mTP**	**0.792**
SP	0.031
other	0.014
**Loc**	**M**
**RC**	**3**

Figure S2. *In silico* prediction of SmsHSP24.1 protein subcellular localization.

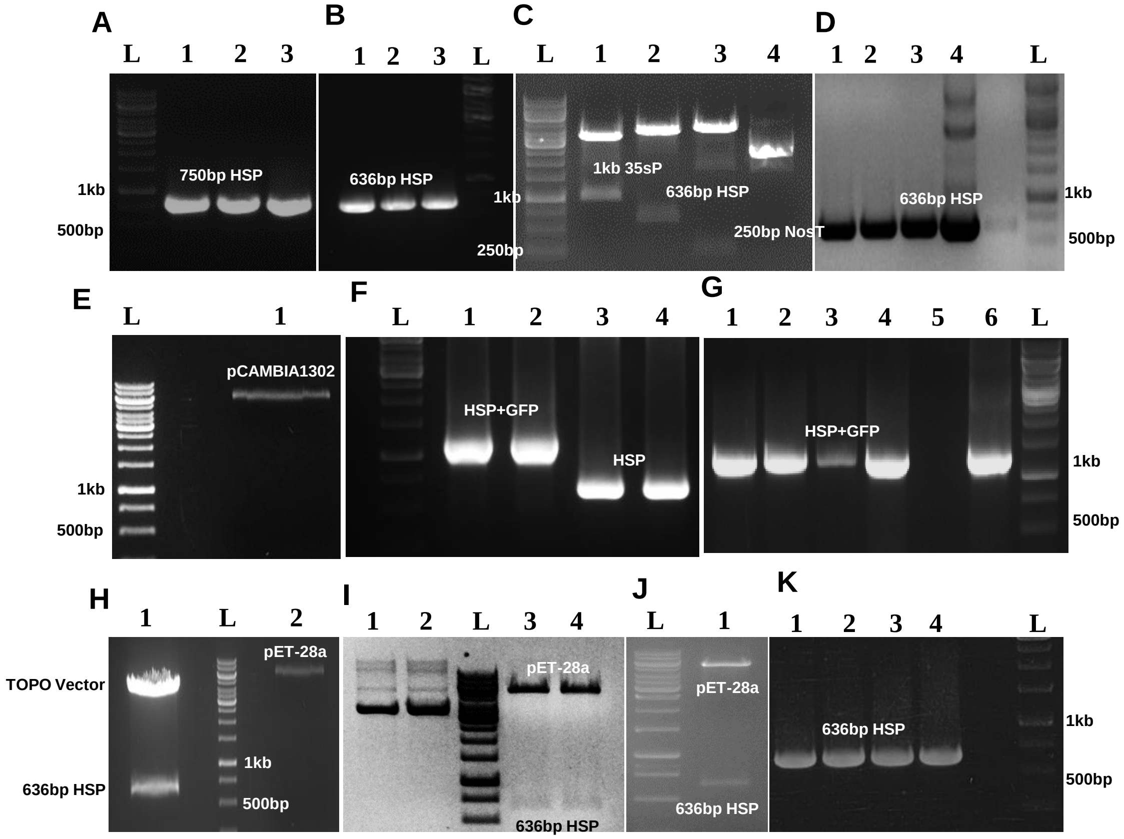

Figure S3. Construction of all expression vectors.

(A, B) Amplification of SmsHSP24.1 coding region from eggplant cDNA by nested PCR, L: represents 1kb DNA ladder, 1, 2, 3: SmsHSP24.1 amplified lane. (C) The SmsHSP24.1, Cauliflower mosaic virus 35s promoter (35SP) and nopaline synthase gene terminator (NosT) regions sub-cloned into Gateway compatible entry vector (pL12R34-Ap) and restriction digestion conformation, 1: Digestion with *Kpn*I and *Nde*I for 1 kb 35s promoter, 2: Digestion with *Nde*I and *Not*I for 636 bp SmsHSP24.1 coding region, 3: Digestion with *Not*I and *Sac*I for 250 bp Nost, 4: EV1 (pL12R34-Ap) undigested plasmid. (D) PCR confirmation of the SmsHSP24.1 expression cassette in Agrobacterium strain EHA105, 1, 2, 3: SmsHSP24.1 amplified lane, 4: (+) positive control. (E) Green fluorescent fusion protein (mGFP) based expression vector construction. Restriction digestion of pCAMBIA1302 vector with *Nco*I and *Spe*I. (F) SmsHSP24.1 and SmsHSP24.1-mGFP fused construct conformation in pCAMBIA1302, 1, 2: SmsHSP24.1-mGFP fused coding region, 3, 4: SmsHSP24.1 coding region amplification. (G) PCR confirmation of the SmsHSP24.1 expression cassette in Agrobacterium strain EHA105, 1, 2, 3, 4, 5: SmsHSP24.1 amplified lane, 6: (+) positive control. (H) Construction of pET28a::SmsHSP24.1 expression vector for recombinant protein production, (H, J) Restriction digestion of pET-28a (+) and TOPO vector containing SmsHSP24.1, 1: 636 bp SmsHSP24.1 from TOPO vector, 2: pET-28a (+) vector. Later SmsHSP24.1 gene conformation in pET-28a (+) vector. (K) PCR confirmation of the SmsHSP24.1 expression cassette in *E. coli* BL21 (DE3) cell.

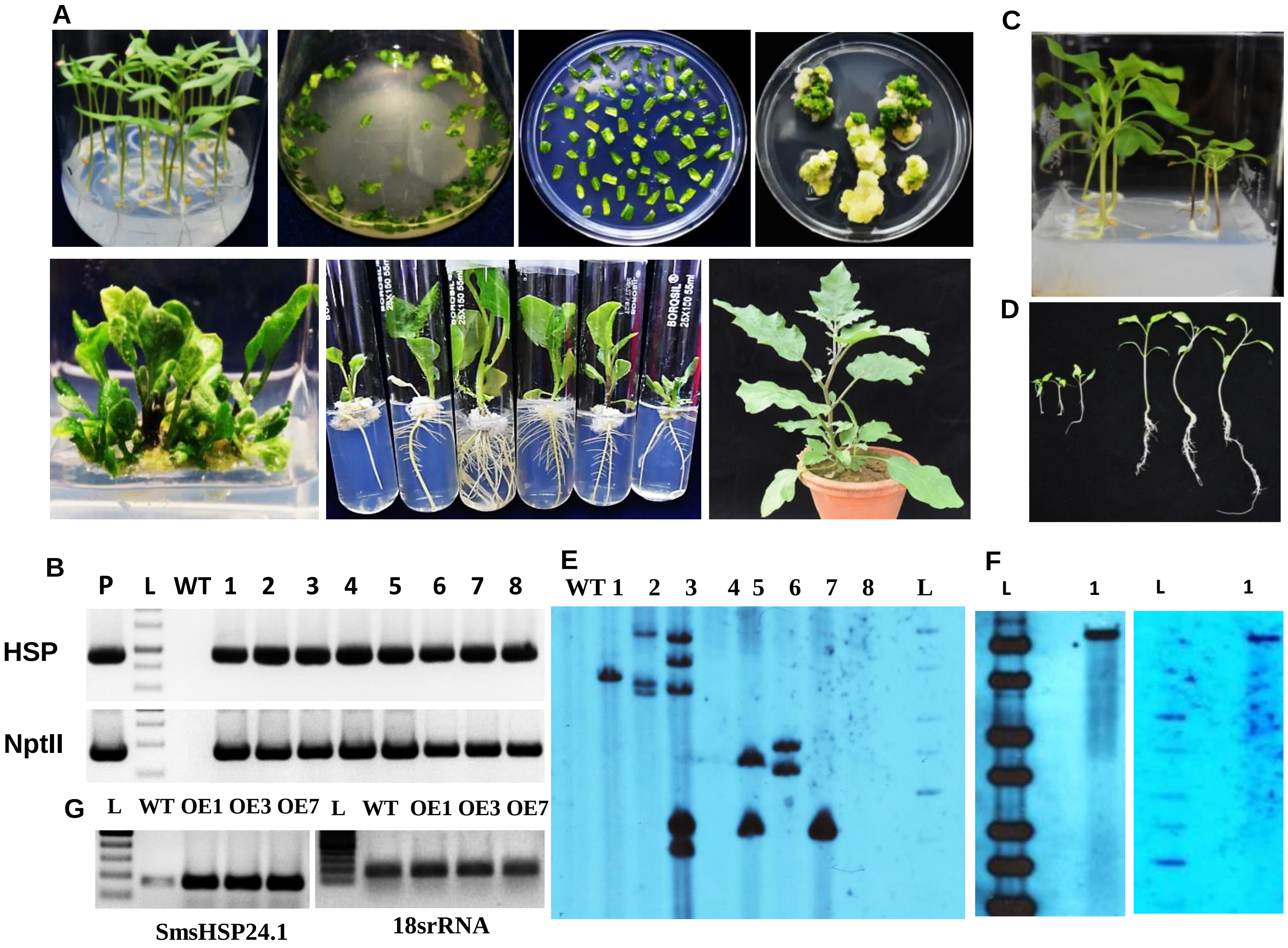

Figure S4. Agrobacterium mediated transformation and Copy number determination of SmsHSP24.1 gene in eggplant genome.

(A) Cotyledonary leaf infected with EHA105 Agrobacterium strain harboring SmsHSP24.1 expression cassette, Initiation of shoot regeneration and multiple shoot development. *In vitro* root formation of the regenerated shoots. Acclimatization and establishment of putative transgenic plantlets. (B) PCR amplification of SmsHSP24.1+NosT terminator and kanamycin (NptII) genes using specific primers in wild type (WT) and eight positive T2 transgenic lines (1-8). L: 1Kb DNA ladder, (P): Positive PCR control. (C, D) Transgenic overexpressed lines were selected under 100 mg/l kanamycin. (E) Southern blot analysis of WT and eight T2 transgenic lines using SmsHSP24.1 cDNA specific probes. (F) Copy number determination by Southern blot analysis, L: 1kb DNA ladder, 1: NdeI, 2: BamHI. (G) Semi quantitative RT-PCR of SmsHSP24.1 transcript in transgenic lines (OE-1, 3, and 7) compared to WT. House keeping gene, 18s rRNA was used as a reference gene (middle panel).

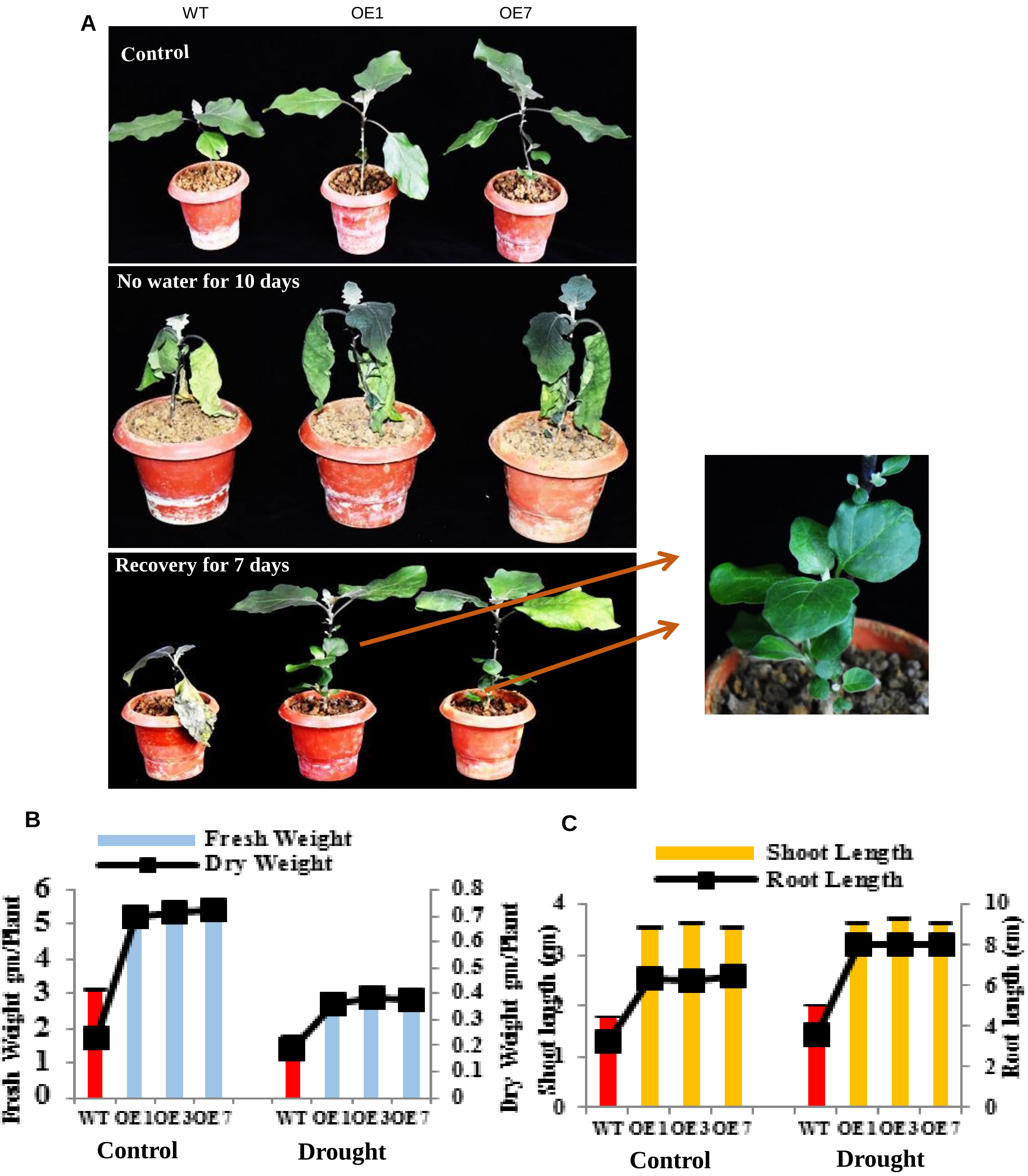

Figure S5. Growth physiology of transgenic eggplant lines overexpressing SmsHSP24.1 protein under drought condition.

(A) Growth parameters of the transgenic as well as WT eggplant lines under drought stress by withholding water for 10 days and after one week of re-watering. (B,C) Total fresh weight, dry weight, root and shoot length were significantly higher in all transgenic seedlings compared to WT line.

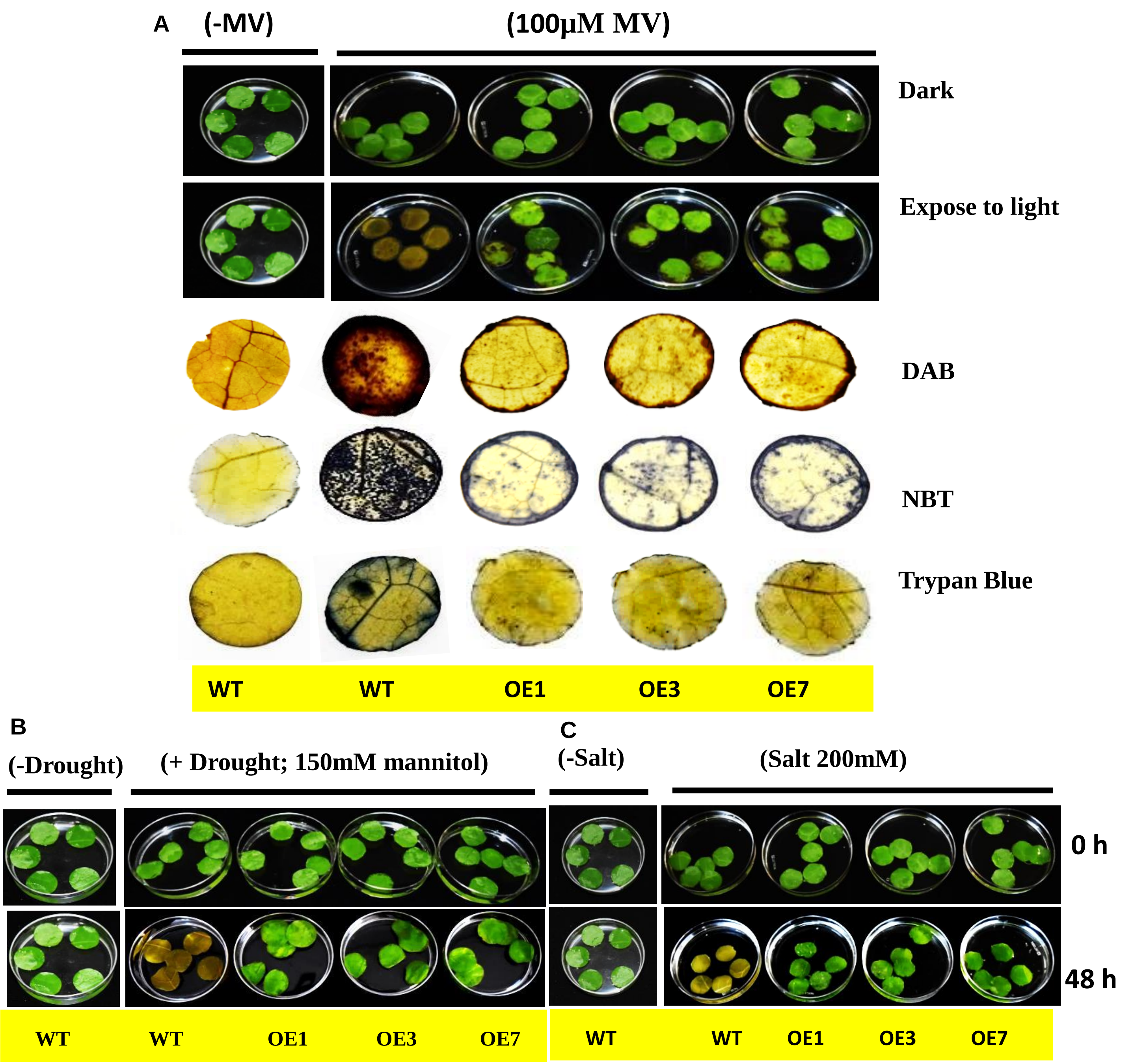

Figure S6. Assessment of cellular damage of transgenic eggplants lines overexpressing SmsHSP24.1 protein in response to methyl viologen (MV), 200 mM NaCl and 150 mM mannitol induced oxidative stress.

(A) Leaf discs were pre-incubated with 10 µM methyl viologen (MV; paraquat) and then exposed to sunlight subsequently relative H_2O_2 and O^{2-} accumulation and death cells were assessed by histochemical staining. (B,C) Leaf discs were incubated for 48 hours in 200 mM NaCl as salt and 150 mM Mannitol as drought stress.

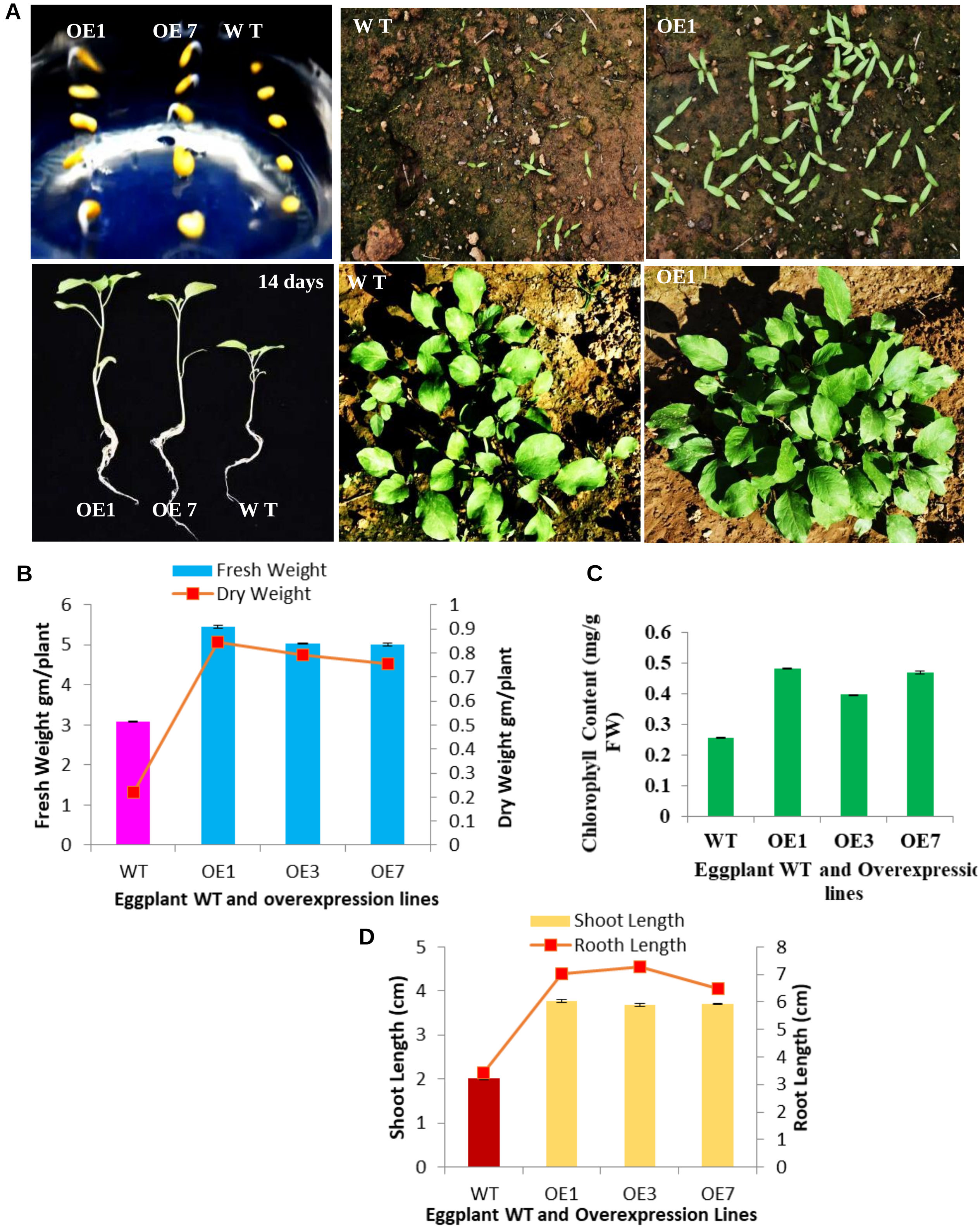

Figure S7. Germination rate and growth physiology of WT and transgenic eggplant lines under normal environmental growth condition.

(A) Seed germination and seedling vigor of transgenic lines in *in-vitro* and field grown conditions compared to WT plants. (B-D) Total fresh weight, dry weight, root length, shoot length and chlorophyll content was significantly higher in all transgenic seedlings compared to WT. Data are mean ± SD (n = 3). The letters a, b, c and others indicate significant differences (P <0.05). (h) Growth phenotypes of transgenic and WT lines under field condition with normal watering.

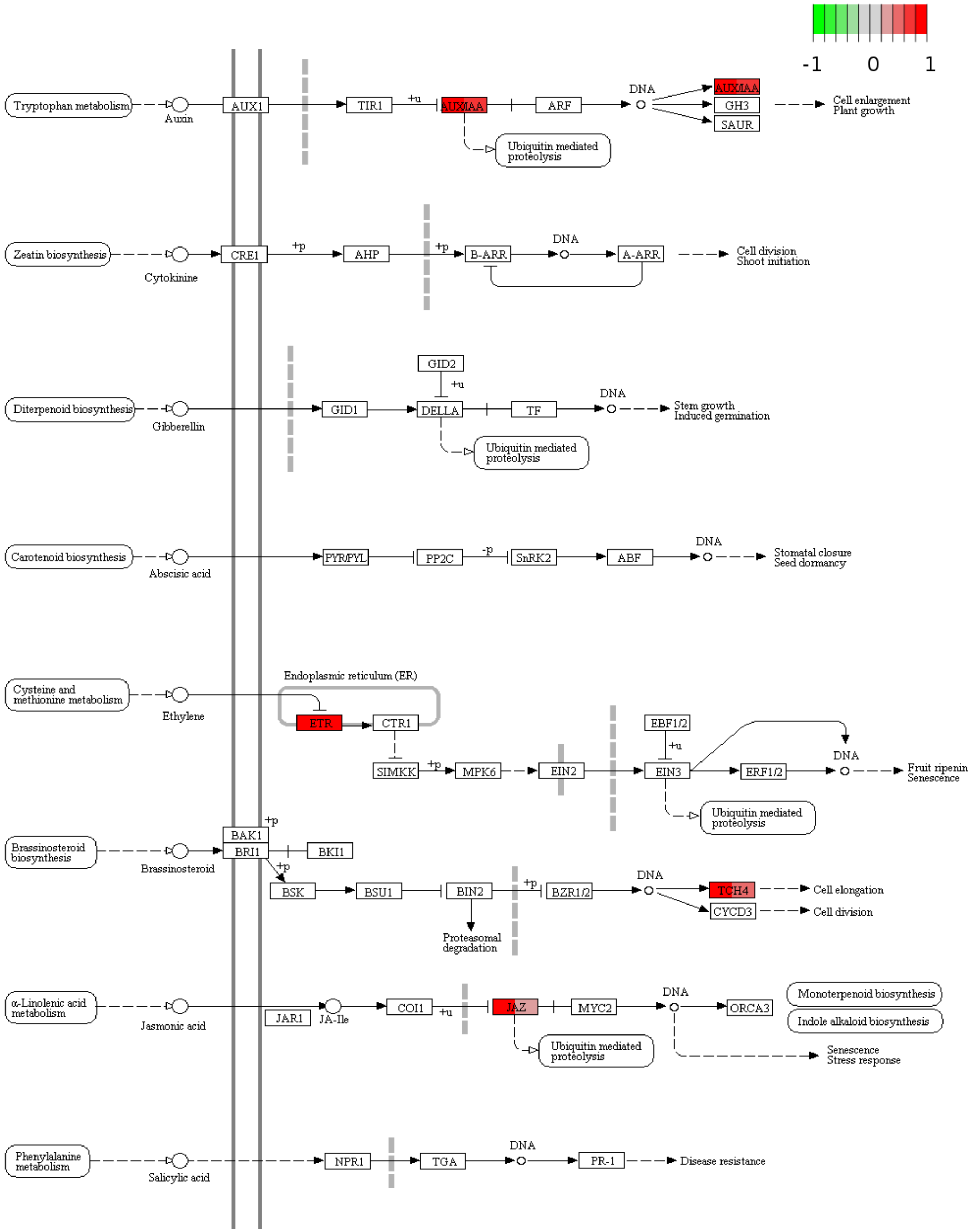

Figure S8. Plant hormone signalling pathways significantly affected in SmHSP24.1overexpressed transgenic (OE) plants as compared to wild type (WT).

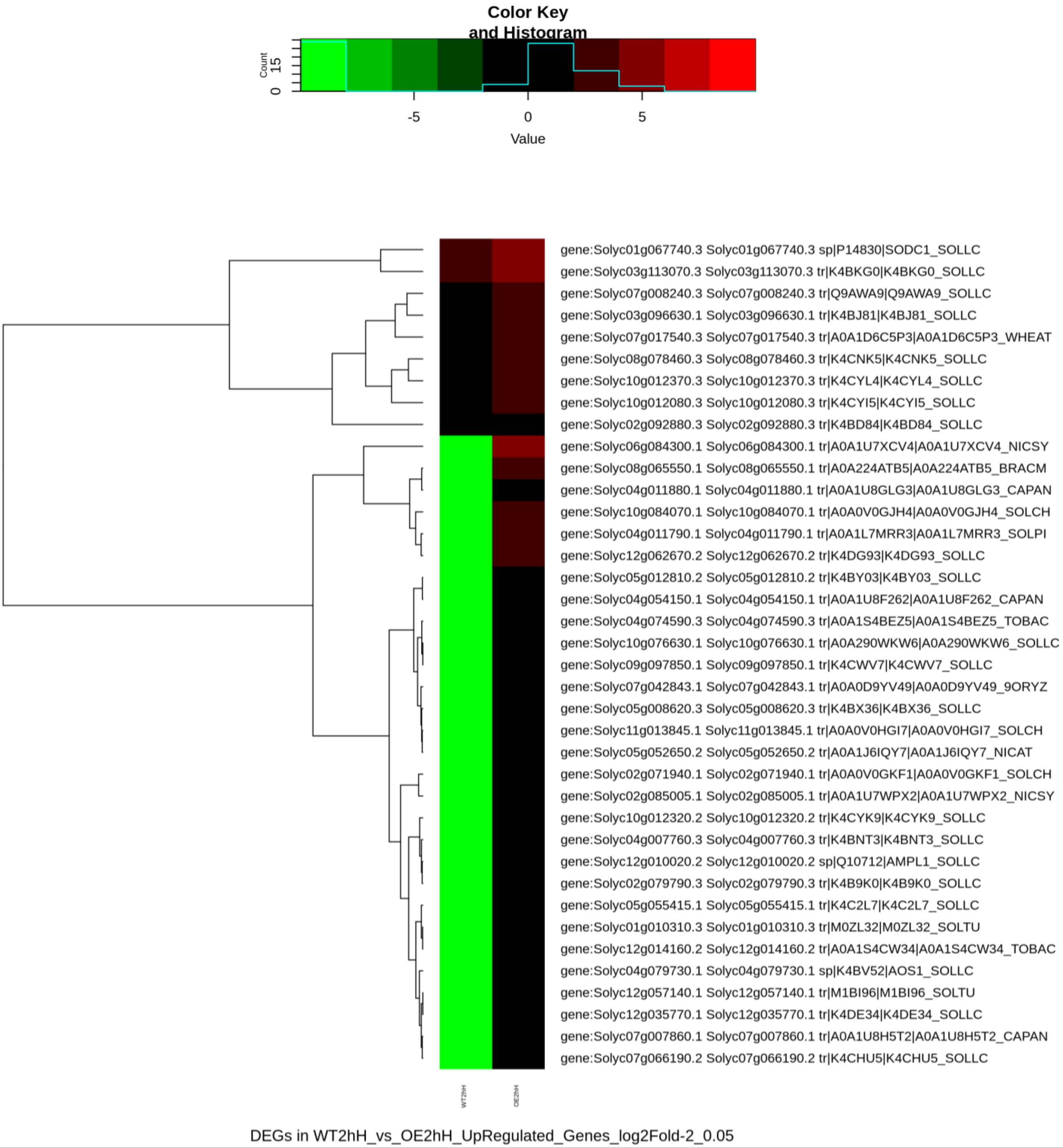

Figure S9. Heatmap generated from differentially expressed up-regulated genes in OE2h vs WT2h lines.

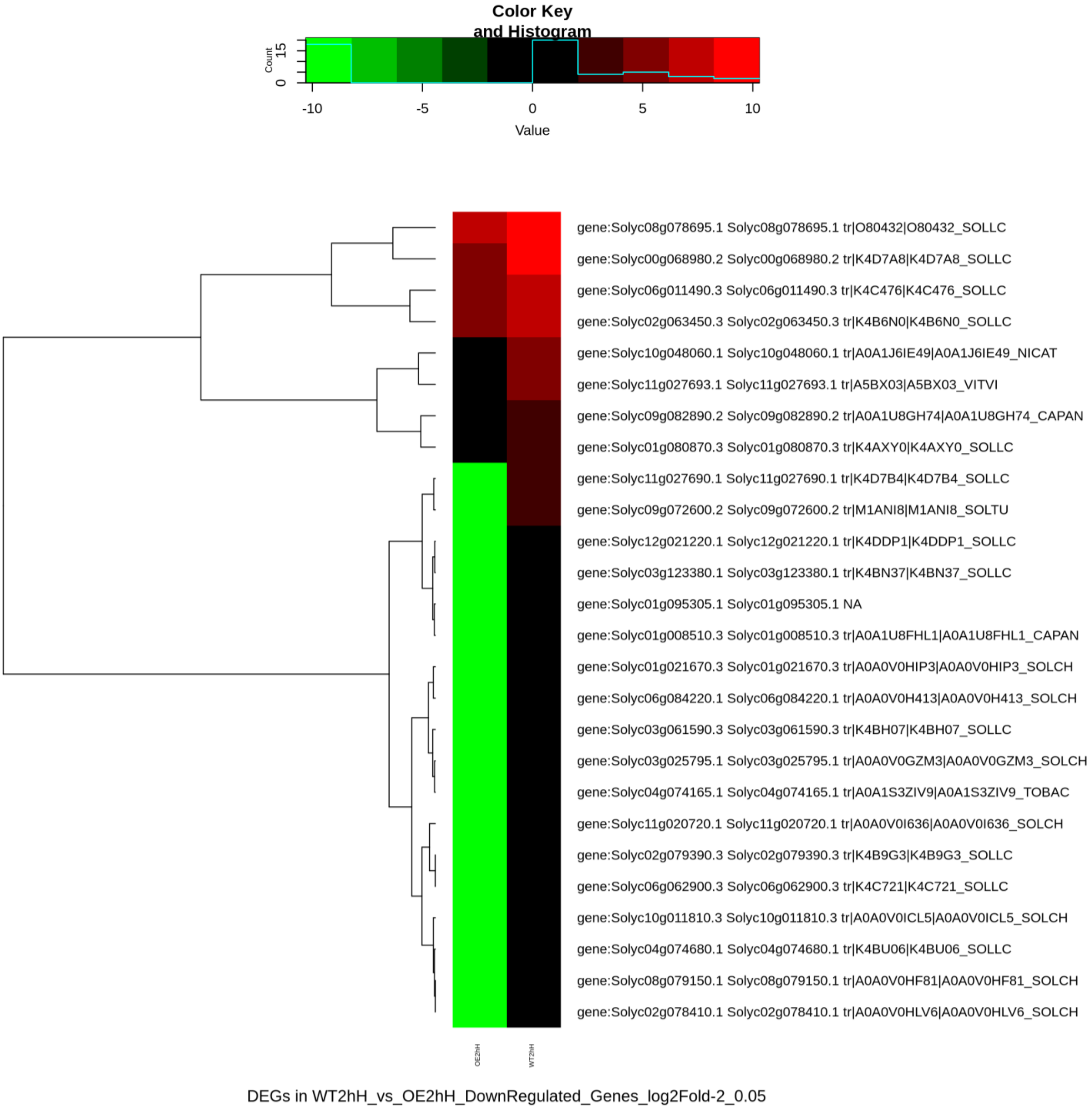

Figure S10. Heat map of differentially expressed (DEGs) down-regulated genes in SmHSP24.1overexpressed transgenic (OE) and wild type (WT) lines under stress conditions.

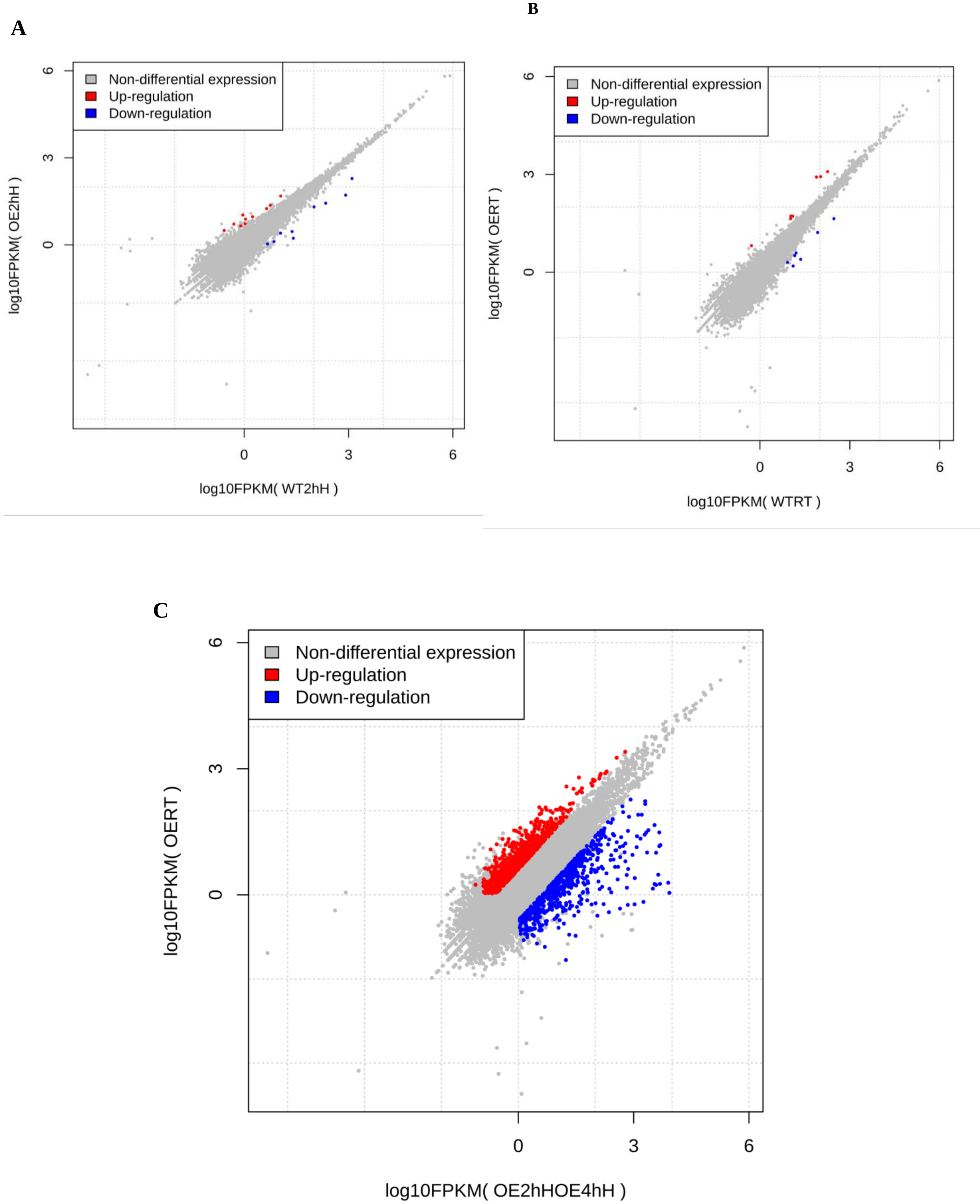

Figure S11. (A,B,C) Scatter plot of FPKM in overexpressed (OE) and wild type (WT) lines under control and heat stress.
