# Supplementary material for: Mitochondria-Targeted SmsHSP24.1 Overexpression Stimulates Early Seedling Vigor and Stress Tolerance by Multi-Pathway Transcriptome-Reprogramming
Source: Front Plant Sci. 2021 Nov 23;12:741898. doi: 10.3389/fpls.2021.741898 (PMC8649800; doi:10.3389/fpls.2021.741898)
Supplement: Supplementary file 2 [file Data_Sheet_2.PDF]

Table S1: Down regulated DGEs in Overexpressed OE lines compared to wild type (WT) eggplant.

Gene symbol	log2(fold change)	Protein name
1. Hydrolase activity [GO:0016787]		
Solyc05g055290.3	-2.52001	Uncharacterized protein
Solyc08g077210.3	-2.0285	Inositol-1,4,5-triphosphate-5-phosphatase
Solyc09g010940.3	-2.19575	Uncharacterized protein
Solyc09g065890.3	-3.9333	Uncharacterized protein
Solyc09g075890.3	-8.22641	Uncharacterized protein
Solyc09g091050.3	-2.70169	Uncharacterized protein
Solyc11g020720.1	-2.34042	Putative hydrolase-like
Solyc12g096660.2	-2.56891	Uncharacterized protein
2. Nucleic acid binding [GO:0003676]		
Solyc01g096750.1	-3.53618	AGO4D
Solyc02g021060.3	-2.58953	Polyprotein, putative
Solyc03g058970.3	-2.19536	Retrovirus-related Pol polyprotein from transposon TNT 1-94
Solyc05g056360.2	-2.4012	Uncharacterized protein
Solyc08g065820.2	-5.9472	Uncharacterized protein
Solyc09g013120.3	-2.4022	Uncharacterized protein
Solyc10g055620.2	-2.54615	Putative ovule protein
Solyc11g071690.2	-2.40188	Uncharacterized protein
3. Unfolded protein binding [GO:0051082]		
Solyc01g106210.3	-4.57933	Uncharacterized protein
Solyc01g106260.3	-4.74412	Uncharacterized protein
Solyc03g007890.3	-7.90436	Uncharacterized protein
Solyc04g009770.3	-2.64908	Uncharacterized protein
Solyc04g081570.3	-2.19139	Putative endoplasmin-like
Solyc05g055160.3	-2.11419	Uncharacterized protein
Solyc06g036290.3	-8.68791	Putative heat shock protein 83-like
Solyc07g047790.3	-3.05419	Uncharacterized protein
Solyc11g020040.2	-10.7598	Uncharacterized protein
Solyc11g071830.2	-7.29679	DnaJ protein isogeny ANJ1
Solyc12g015880.2	-2.30258	Molecular chaperone Hsp90-1
4. Ubiquitin protein ligase activity [GO:0061630]		
Solyc01g050040.3	-3.12777	Uncharacterized protein
Solyc02g065540.2	-3.6894	Uncharacterized protein
Solyc03g114160.1	-2.15148	RING-type E3 ubiquitin transferase
Solyc05g005670.1	-2.9996	RING-type E3 ubiquitin transferase

Solyc05g010650.3	-2.08492	RING-type E3 ubiquitin transferase
Solyc06g083150.3	-3.5218	Uncharacterized protein
5. Cell wall organization or biogenesis [GO:0071554]		
Solyc03g007120.3	-3.04653	Uncharacterized protein
Solyc03g093130.3	-6.73563	Xyloglucan endotransglucosylase/hydrolase
Solyc07g006850.2	-3.27768	Xyloglucan endotransglucosylase/hydrolase
Solyc09g092520.3	-2.20259	Xyloglucan endotransglucosylase/hydrolase
Solyc12g017240.2	-6.35822	Xyloglucan endotransglucosylase/hydrolase
6. Carbohydrate metabolic process [GO:0005975]		
Solyc01g058140.3	-2.16348	Putative beta-glycosidase-like
Solyc02g063220.3	-2.59015	Mannose-6-phosphate isomerase
Solyc11g068440.2	-2.03865	Uncharacterized protein
Solyc12g008840.2	-2.57425	Beta-galactosidase
Solyc12g098810.2	-3.62086	Uncharacterized protein

Table S2: List of oligonucleotide primer sequences used for this work

Target sequence	Primer	Sequence (5’ - 3')	Amplicon size (bp)
SmHSP24.1	SmHSP24.1_F1	TCGGCAAGTTCATACAGCTGCA	750
	SmHSP24.1_R1	ATCACTGCAGACCAACCTAGCT	
SmHSP24.1	SmHSP24.1_F2	GCACATATGGCAACTTCACTTGCTCTC	636
	SmHSP24.1_R2	ATTGCGGCCGCTCACTCAATTTGAACATTG	
SmHSP24.1	SmHSP24.1_qRT_F1	CTCACCACCAAGGAGCGTGAGC	172
	SmHSP24.1_qRT_R1	CTTGTCGAGCCCAGGCATATCC	
SmHSP24.1	SmHSP24.1-GFP_F1	TGCCATGGTGATGGCAACTTCACTTGCTC	636
	SmHSP24.1-GFP_R1	GCACTAGTCTCAATTTGAACATTGAAAAC	
18S rRNA	18S rRNA_qRT_F1	CATTGAGAAATGGCCAGACC	172
	18S rRNA_qRT_R1	AAGACCGGCAACAGGATTC	
35sP	35sP_F1	GGTACCGCGTATTGGCTAGAGCA	1000
	35sP_R1	CATATGTGGAGTGTCTCGAACTTCTTCTTCC	
NosT	NosT_F1	GCGGCCGCCGATCGTTCAAACATTTGGCA	250
	NosT_R1	GAGCTCAATTCCCGATCTAGTAACATAGATG	
NptII	NptII_F1	TGGCTGCTATTGGGCGAAGTGC	480
	NptII_R1	CCATTCGCCGCCAAGCTCTTCA	
HSP:GFP fused	HSP Fused F	TGCCATGGTGATGGCAACTTCACTTGCTC	1189
	GFP_R1	TGATAATGATCAGCGAGTTGC	
NADH-ubiquinone oxidoreductase	NdhF_qRT_F1	GGATATTCGCCAGCTAAAAGCC	172
	NdhF_qRT_R1	TGGACCAAAAACAAGCAAGAGG	
PepA	PepA_qPCR_F1	TCACACTTGCTGATGCGTTG	170
	PepA_qPCR_R1	TTCATCGCTTGCCTCAGAAG	
SOD1	SOD1_qPCR_F1	AGCCGTAGTTGTTCATGCTG	172
	SOD1_qPCR_R1	AGGACAAACCACGCTTTTGC	
SOD2	SOD2_qPCR_F1	CGTGGCTGAAGCAACAATTG	163
	SOD2_qPCR_R1	AGCTCGTGAACTACAAGTGC	
SAUR	SAUR _qPCR_F1	AACAAGAGGCTGAGTTGTGC	170
	SAUR _qPCR_R1	TGTTTTGGGGCAATCGGTTC	
Quinol-cytochrome-c oxidoreductase	Cytc_qPCR_F1	ACAAGGACCCAACTTGAACG	107
	Cytc_qPCR_R1	TATTCTCTCCCCAGGTAACAGC	
CAT	CAT_qPCR_F1	ATTCTGGTGCTCCTGTTTGG	112
	CAT_qPCR_R1	ACGTTCCCTGTCAAAGTTGG	
APX	APX_qPCR_F1	CCTTTTCCGCACTTCATACC	116
	APX_qPCR_R1	TGGATCCAAGGCTACAGTGAG	

Table S3: RNAseq reads alignment summary

Sample Name	Total Read Count	Read Count after rRNA removal	QC Pass %	Aligned %	Unaligned %
OERT	560,98,118	548,17,600	97.72	65.6	34.4
WTRT	835,59,126	808,99,814	96.82	65.27	34.73
OE2hTreated	735,03,888	721,77,768	98.2	56.88	43.12
WT2hTreated	824,94,878	719,89,620	87.27	57.76	42.24
